# Synthesis and preclinical evaluation of a novel fluorine-18 labeled small-molecule PET radiotracer for imaging of CXCR3 receptor in mouse models of atherosclerosis

**DOI:** 10.1186/s13550-023-01017-x

**Published:** 2023-07-13

**Authors:** Santosh R. Alluri, Yusuke Higashi, Ashley Berendzen, Laurel A. Grisanti, Lisa D. Watkinson, Kamlendra Singh, Timothy J. Hoffman, Terry Carmack, Elizabeth A. Devanny, Miles Tanner, Kun-Eek Kil

**Affiliations:** 1 University of Missouri Research Reactor, University of Missouri, 1513 Research Park Drive, Columbia, MO 65211 USA; 2 Department of Medicine, Tulane University School of Medicine, New Orleans, LA 70112 USA; 3 Research Service, Harry S. Truman Memorial Veterans’ Hospital, Columbia, MO 65201 USA; 4 Department of Biomedical Sciences, College of Veterinary Medicine, University of Missouri, Columbia, MO 65211 USA; 5 Bond Life Sciences Center, University of Missouri, Columbia, MO 65211 USA; 6 Department of Veterinary Medicine and Surgery, College of Veterinary Medicine, University of Missouri, Columbia, MO 65211 USA; 7 Present Address: Department of Radiology and Biomedical Imaging, Yale University, New Haven, CT 06519 USA

**Keywords:** Atherosclerosis, CXCR3 chemokine receptor, PET imaging, Inflammation, Small-molecule radiotracer

## Abstract

**Background:**

CXCR3 is a chemokine receptor and is expressed in innate and adaptive immune cells. It promotes the recruitment of T-lymphocytes and other immune cells to the inflammatory site in response to the binding of cognate chemokines. Upregulation of CXCR3 and its chemokines has been found during atherosclerotic lesion formation. Therefore, detection of CXCR3 by positron emission tomography (PET) radiotracer can be a useful tool for detecting the development of atherosclerosis in a noninvasive manner. Herein, we report the synthesis, radiosynthesis, and characterization of a novel fluorine-18 (F-18, ^18^F) labeled small-molecule radiotracer for the imaging of the CXCR3 receptor in mouse models of atherosclerosis.

**Results:**

The reference standard **1** and its precursor **9** were synthesized over 5 steps from starting materials in good to moderate yields. The measured K_i_ values of CXCR3A and CXCR3B were 0.81 ± 0.02 nM and 0.31 ± 0.02 nM, respectively. [^18^F]**1** was prepared by a two-step radiosynthesis with a decay-corrected radiochemical yield of 13 ± 2%, radiochemical purity > 99%, and specific activity of 44.4 ± 3.7 GBq/µmol at the end of synthesis (*n* = 6). The baseline studies showed that [^18^F]**1** displayed high uptake in the atherosclerotic aorta and brown adipose tissue in Apolipoprotein E (ApoE) knockout (KO) mice fed with a high-fat diet over 12 weeks. The uptake of [^18^F]**1** in these regions was reduced significantly in self-blocking studies, demonstrating CXCR3 binding specificity. Contrary to this, no significant differences in uptake of [^18^F]**1** in the abdominal aorta of C57BL/6 control mice fed with a normal diet were observed in both baseline and blocking studies, indicating increased CXCR3 expression in atherosclerotic lesions. Immunohistochemistry studies demonstrated that [^18^F]**1**-positive regions were correlated with CXCR3 expression, but some atherosclerotic plaques with significant size were not detected by [^18^F]**1**, and their CXCR3 expressions were minimal.

**Conclusion:**

[^18^F]**1** was synthesized with good radiochemical yield and high radiochemical purity. In PET imaging studies, [^18^F]**1** displayed CXCR3-specific uptake in the atherosclerotic aorta in ApoE KO mice. [^18^F]**1** visualized CXCR3 expression in different regions in mice aligned with the tissue histology studies. Taken together, [^18^F]**1** is a potential PET radiotracer for imaging CXCR3 in atherosclerosis.

**Supplementary Information:**

The online version contains supplementary material available at 10.1186/s13550-023-01017-x.

## Introduction

Atherosclerosis is a chronic inflammatory disease of the artery wall and is characterized by ectopic fat deposition within the arterial wall, and the lesion development is mediated by a vicious cycle of inflammatory response and cell death [1]. Chemokines and chemokine receptors are key signal axis that mediates the leukocyte migration into the artery wall, thereby promoting the progression of atherosclerotic lesions [2]. Chemokines are a large family of small peptides that signal through cell surface receptors and induce a chemotactic response in the recipient cells [3, 4]. The two major subfamilies of chemokines are CC chemokines and CXC chemokines, named after their primary structures, wherein CC chemokines have two *N*-terminal cysteines next to each other and CXC chemokines have two *N*-terminal cysteines separated by one amino acid. Each chemokine has single or multiple chemokine receptors as its partners and vice versa. CXCR3 is one of the six CXC chemokine receptors and is highly expressed on different types of leukocytes, including CD4^+^ type-1 helper T cells (Th1), natural killer (Nk) cells, and monocytes under inflammation and infection conditions [4]. In fact, the upregulation of CXCR3 and its chemokines has been found in a variety of inflammatory diseases such as multiple sclerosis, arthritis, and atherosclerosis [5]. CXCR3 inhibition is emerging as a promising therapeutic strategy for inflammatory disorders [5].

CXCR3 binds with three CXC chemokines, CXCL9, CXCL10, and CXCL11, and mediates intracellular signalling [3, 4]. These chemokines are secreted by macrophages and endothelial cells during the formation of atherosclerotic lesions [6], thereby playing integral roles in the recruitment of CXCR3-expressing proinflammatory leukocytes to the lesion and thus promoting inflammation and, ultimately, atherosclerosis [6–8]. It has been reported that the atherosclerotic lesion formation was shown to decrease with the deletion of either CXCR3 or its ligand CXCL10 in apolipoprotein E (ApoE) knockout (KO) mice, a widely used rodent model of atherosclerosis [9, 10].

**Scheme 1:**
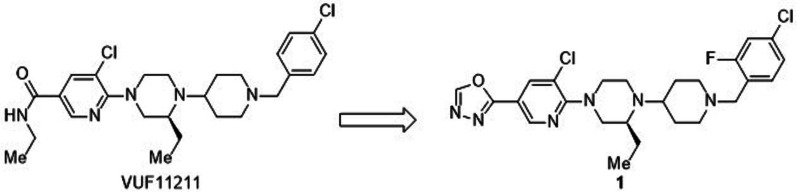
Schematic example of the current CXCR3 antagonists [11–13]

Over the last two decades, an extensive array of small-molecule CXCR3 inverse agonists have been identified from high-throughput, virtual screening approaches [14]. Initially, Amgen and Neurocrine Biosciences reported 8-azaquinazolinone-based molecules as CXCR3 antagonists with nanomolar affinities [14, 15], then pyridyl-piperazinyl-based molecules with the highest affinities and selectivity were disclosed by Merck Research Laboratories [14]. The latter is taken into consideration in this study. Scheme 1 depicts the strategic and consecutive development of various antagonists based on a pyridyl-piperazinyl-piperidine scaffold flanked by a benzyl unit and a polar group such as amide or substituted amide. A representative compound of this class is VUF11211 (K_d_ = 0.65 nM) which antagonizes CXCR3-chemokines action [16, 17]. Despite the availability of such highly potent CXCR3 antagonists, the development of small-molecule radioligands is limited. Recently, tritium (H-3, ^3^H) labeled VUF11211 ([^3^H]VUF11211) was assessed in vitro*,* and the results suggested that it has a high affinity for human CXCR3 and has fast binding kinetics [17]. The positron emission tomography (PET) imaging probes of such compounds can provide sensitive Information that enables the investigation of pathophysiological processes of various inflammatory disorders, including atherosclerosis.

Various radiotracers for chemokine receptors, mostly labeled with radiometal, have previously been developed to visualize the inflammation mechanism involved in atherosclerosis [18]. [^68^Ga]Ga-Pentixafor and [^64^Cu]Cu-DOTA-DAPTA have successfully imaged inflammatory atherosclerosis via CXCR4 and CCR5 chemokine receptors, respectively [19, 20]. [^18^F]2-Fluorodeoxyglucose ([^18^F]FDG) has shown promise in atherosclerosis imaging, but its use is limited for such imaging because of its non-specific uptake by metabolically active myocardium [21]. To our knowledge, there is no reported PET tracer specific to CXCR3 receptor though its role in atherosclerosis is evident. In this study, we chose compound **1** from the reported CXCR3-specific compound libraries. VUF11211 may not be suitable for PET studies as it lacks a proper labeling site with carbon-11 or F-18, and the ethylamide part of VUF11211 is metabolically labile. The target compound **1** overcomes these disadvantages, and, in addition, its high potent binding affinity (IC_50_ = 0.8 nM) to CXCR3 encouraged us to develop and validate its isotopologue [^18^F]**1** [13]. Herein, we report the synthesis of [^18^F]**1** and its PET validation in ApoE KO mice in comparison to control C57BL/6 mice as a proof-of-principle.

## Results

### Computer-aided modeling (CAM) study

**Fig. 1:**
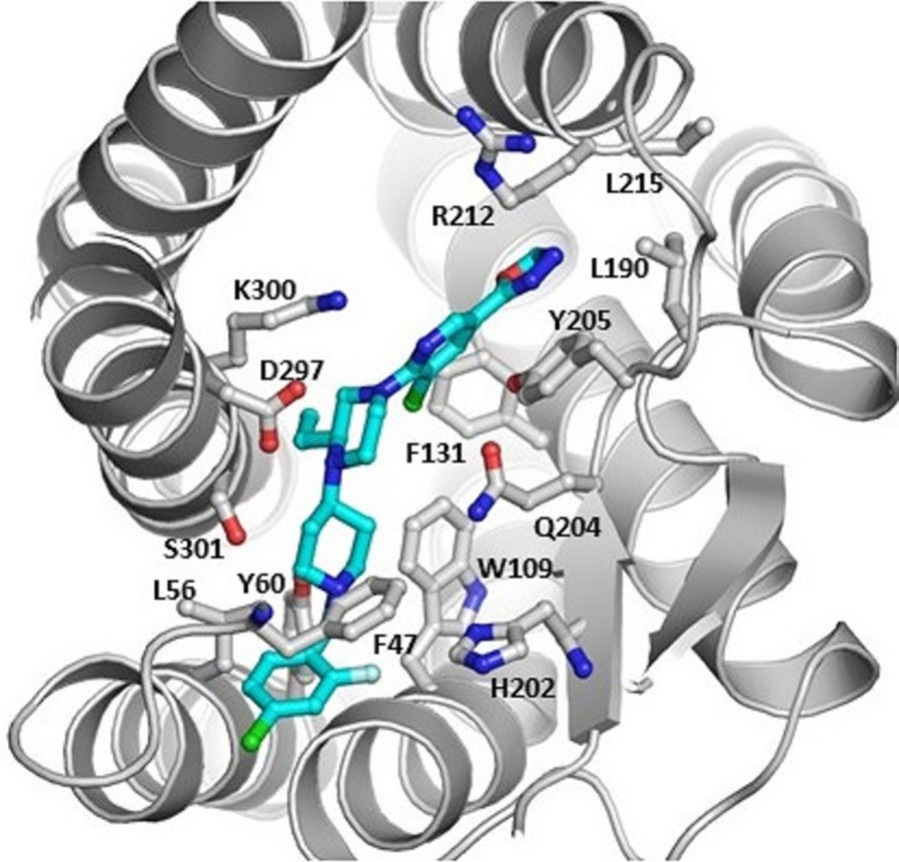
The cartoon of the docking model for compound **1** that has 14 interactions with the amino acids of CXCR3

Figure 1 shows the results of the CAM experiment. The CAM work shows that **1** has interactions with 14 amino acid residues. It has total 6 π–π interactions with Phe 47, Tyr 60, Trp 109, Phe 131, His 202, and Tyr 205 and 3 hydrophobic interactions with Leu 56, Leu 190, and Leu 215. It also has 5 hydrogen bonds with Glu 204, Arg 212, Asp 297, Lys 300, and Ser 301. The resultant docking score of **1** was -5.124. Additional hydrophobic interactions contribute to the binding of **1** with CXCR3. The residues involved in hydrophobic interactions include W109, A113, L190, and the carbon chain of R212. The primary stability of the receptor-ligand complex appears to come from the abovementioned hydrophobic and π–π interactions.

### Chemistry

**Scheme 2:**
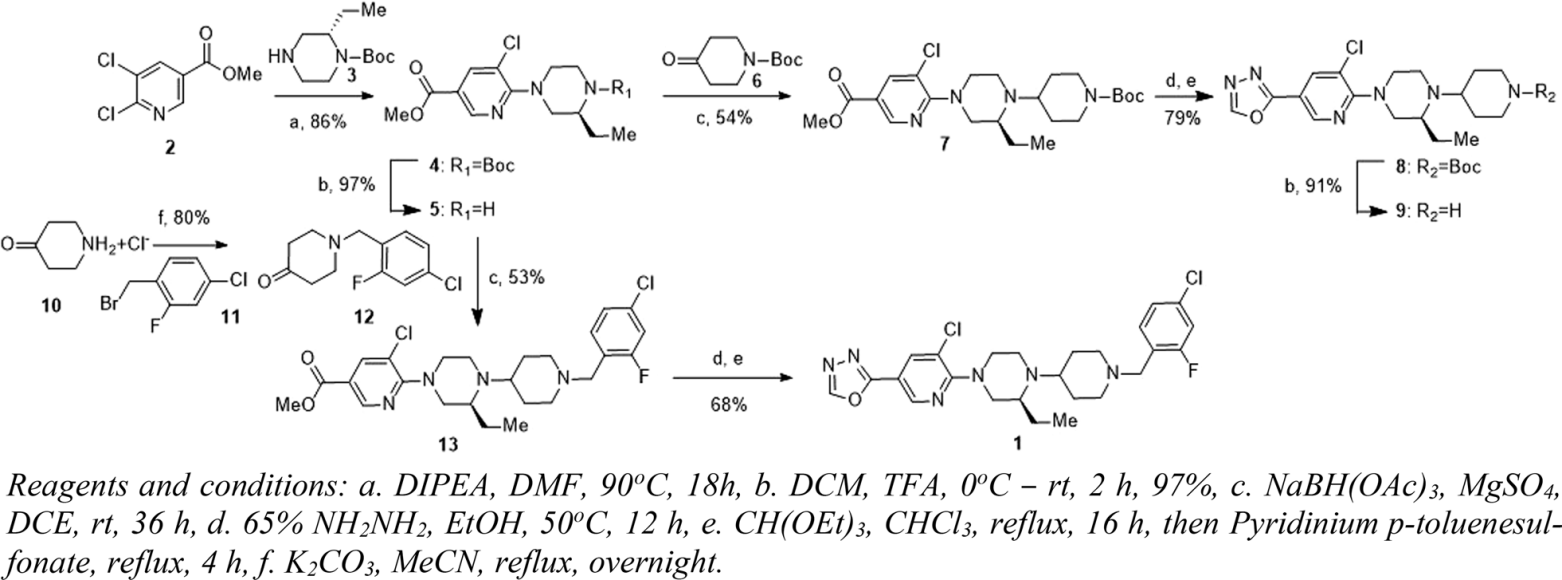
Synthetic scheme for nonradioactive standard **1** and its precursor **9**

Synthesis of **1** and its radiolabelling precursor **9** are shown in Scheme 2. The syntheses were carried out using reported methods with modifications [15]. At first, compound **5** was synthesized by heating **2** and (S)-*N*-Boc-3-ethylpiperazine (**3**) under basic conditions, followed by -Boc removal using trifluoroacetic acid (TFA). Compound **5** was used as a common intermediate to make **1** and its precursor **9**. Reductive amination of **5** with 1-Boc-piperidin-4-one (**6**) afforded **7** and similarly with **12** obtained by benzylation of piperidin-4-one (**10**) gave **13**. The carboxylic ester derivatives **7** and **13** were then treated with hydrazine followed by triethyl orthoformate and pyridinium *p*-toluenesulfonate to obtain the intermediate compound **8** and reference standard **1**, respectively. Finally, precursor **9** was obtained from **8** upon Boc deprotection. Reference standard **1** was prepared as a hydrochloride (HCl) salt. After **1** was dissolved in the ethanol (EtOH), an excess amount of conc. HCl was added to the solution. The whole solution was further dried in a stream of nitrogen until a white solid was left behind. The residual solvent was further removed in vacuo and used as a blocking agent.

### Radiochemistry

**Scheme 3:**
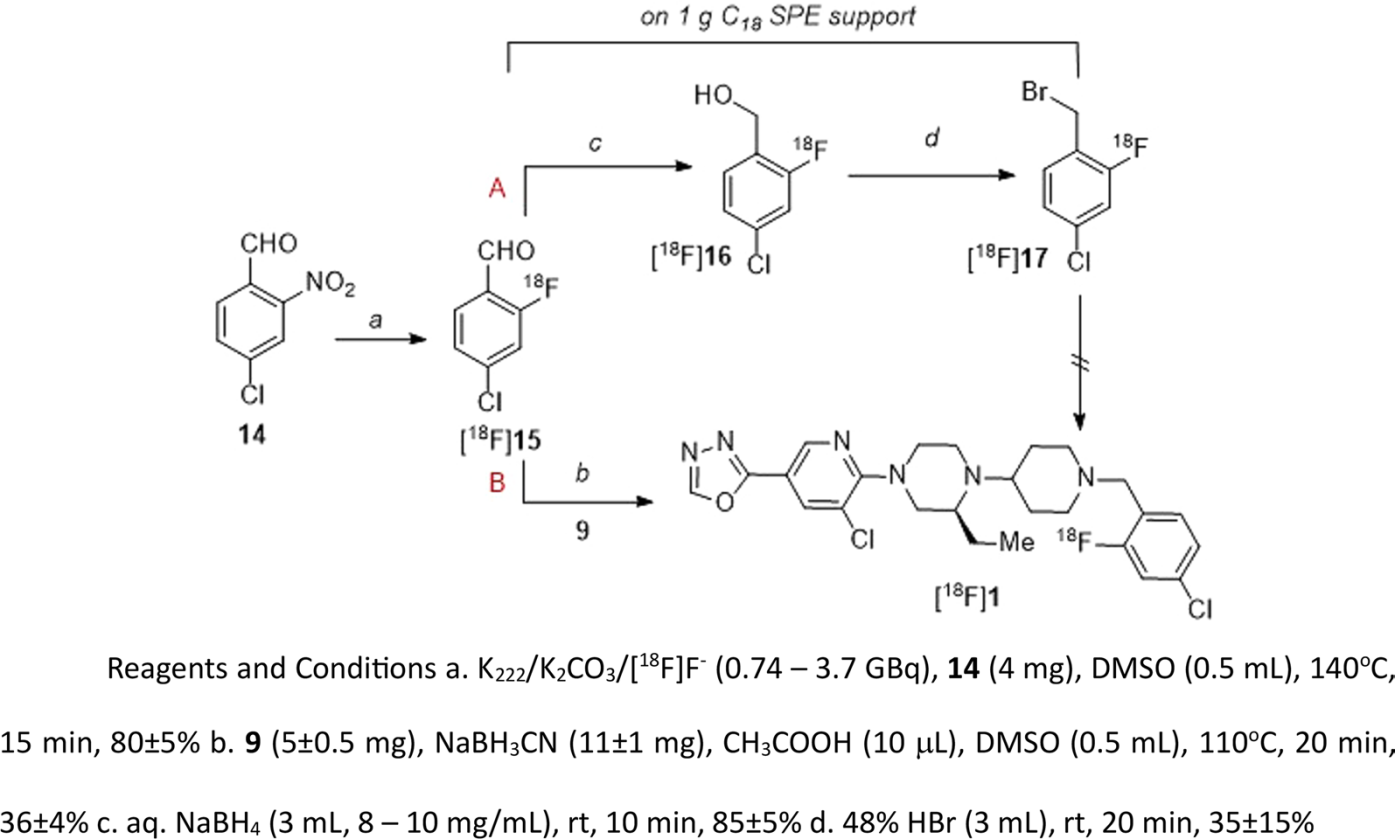
(A) Attempted four-step synthesis and (B) One-pot two-step radiosynthesis of [^18^F]**1**

Initially, literature method [22] for the automated preparation of F-18 labeled benzylhalides has tempted us to exploit the strategy to prepare [^18^F]**1**. Scheme 3A depicts an attempted 4-step radiosynthesis approach that involves (i) nucleophilic substitution of 4-chloro-2-nitrobenzaldehyde (**14**) with [^18^F]fluoride for [^18^F]**15** (ii) reduction to alcohol [^18^F]**16**, (iii) bromination to [^18^F]**17**, (iv) heating bromide with **9** under basic conditions to make [^18^F]**1**. We have used a semi-automated setup to prepare the desired radiolabelled intermediates and finally [^18^F]**1**. The intermediate [^18^F]**15** was trapped on a C_18_ solid phase extraction (SPE) cartridge (1 g), and the subsequent reduction and halogenation were performed on SPE support. At first, an aqueous solution of sodium borohydride (NaBH_4_, 3 mL, 8–10 mg/mL) was passed slowly through support over 10 min for converting [^18^F]**15** to [^18^F]**16,** and > 85% radiochemical conversion (RCC) based on radio-thin layer chromatography (TLC) was noticed (*n* ≥ *10*). After a rinse with water (5 mL) and a flush with nitrogen, 48% bromic acid (HBr) or 33% HBr/acetic acid (AcOH, 3 mL) was passed through the SPE support over 15 to 20 min for converting [^18^F]**16** to [^18^F]**17** and moderate RCC (35 ± 10%, radio-TLC) was noticed at this step (*n* ≥ *8*). Neither an increase in reaction time nor the pre-heated (50°C, 5–10 min) HBr was not useful in enhancing RCC to [^18^F]**17**. Considerable damage to C_18_ support was observed when warm HBr was used. At this stage, after a rinse with water (5 mL) and a flush with nitrogen, the radiolabelled intermediates mixture trapped in the C_18_ SPE cartridge was eluted with *N,N*-dimethylformamide (DMF) or acetonitrile (MeCN, 2 mL) and passed through an anhydrous sodium sulfate (Na_2_SO_4_) cartridge into a second reaction vial that already contained **9** (~ 3 mg) and potassium carbonate (K_2_CO_3_, ~ 10 mg) or diisopropylethylamine (DIPEA, ~ 10 μL). The vial was then stirred at 100°C for 20–30 min. However, no desired RCC was noticed during different time intervals and temperatures.

Having seen the difficulties associated with this four-step radiosynthesis method, we decided to put efforts towards a one-pot, two-step reductive amination strategy (Scheme 3B) that involved **9** and [^18^F]**15** [23]. This approach rewarded us not only with reduced radiosynthesis time but also with good decay-corrected radiochemical yield (RCYs) and reproducibility. Precursor **9** is mixed with sodium cyanoborohydride (NaBH_3_CN) and AcOH and transferred into the reaction vial that already has [^18^F]**15** and heated at 110 °C for 20 min (RCC 36 ± 4%, *n* ≥ 10).

Table 1 shows the optimization of step 2 towards good RCC of [^18^F]**15** to [^18^F]**1**. Dimethylsulfoxide (DMSO) was found to be the optimal solvent compared to DMF or methanol (MeOH). The addition of AcOH and the high temperature was necessary for an improved RCC. A usual C_18_ SPE workup followed by semi-preparative reverse phase HPLC purification afforded [^18^F]**1** in RCY of 13 ± 2% (*n* = 10) at the end of synthesis (EOS). The semi-preparative HPLC chromatograms of [^18^F]**1** is shown in Additional file 1: Fig. S1(a). The HPLC purification was performed using a Phenomenex Gemini C_18_ Semi-preparative column (5 µm, 250 × 10 mm) equipped with UV (254 nm) and radioactivity detectors by eluting with an isocratic mobile phase composed of 65% MeCN, 5% MeOH, 30% water, and 0.1% triethylamine (NEt_3_) at a flow rate of 5 mL/min. The radioactive [^18^F]**1** was collected between 16 and 17 min and was well-separated from the rest of the UV and radioactivity peaks. After the purification, the collected product was formulated in 10% EtOH/0.9% saline containing 0.5% sodium ascorbate. The total radiosynthesis time was 100 min at the EOS. The final reformulated aliquot was further characterized by HPLC analysis using a Phenomenex Gemini C_18_ analytical column (5 µm, 250 × 4.6 mm) equipped with UV and radioactivity detectors by eluting with the same HPLC solvent at a flow rate of 1 mL/min. The radioactive peak was eluted between 15 and 17 min. The single injection of the final aliquot confirmed that the obtained radioactive product was > 99% radiochemical purity (RCP) with a specific activity of 44.4 ± 3.7 GBq/µmol at the EOS (*n* = 6). The radiotracer was also shown to be stable for more than 10 h. Then, the aliquot was mixed with nonradioactive **1** standard solution and injected into the same HPLC system. The result of the co-injection confirms that the radioactive peak was eluted together with reference standard **1.** (Additional file 1: Fig. S1(b)).

**Table 1:** The table of optimization for step 2 from [^18^F]**15** to [^18^F]**1**

Entry	Solvent	Additive	Temp	RCC (%)^a^
1	MeOH	HOAc	50	< 3
2	MeOH	None	50	–
3	DMF	HOAc	50	5 ± 3
4	DMF	HOAc	110	12 ± 5
5	DMF	None	110	< 5
6	DMSO	None	110	11 ± 5
7	DMSO	HOAc	110	36 ± 4

Conditions: [^18^F]**15** (740–2220 MBq), **9** (5 ± 0.5 mg), NaBH_3_CN (11 ± 1 mg), HOAc (10 μL), 20 min

^a^Based on radio-TLC (0.1% Et_3_N, 10% MeOH in DCM, R_f_ 0.3)

### Cell-binding assay

The equilibrium inhibition constants (K_i_) value of **1** at CXCR3 receptor was measured through competition binding assay using NBI74330 (Tocris Bioscience, Bristol, UK), an antagonist of CXCR3 receptor, [^125^I]CXCL10 (ViTrax Inc., Placentia, CA) and CXCR3 transfected human embryonic kidney (HEK) 293 cells. CXCR3 has two major variants, CXCR3A and CXCR3B, with distinctive biological roles [24], so two types of HEK293 cells containing each CXCR3 variant were prepared. Before the cell-binding assay, two cDNA constructs containing CXCR3 transcript variant A (CXCR3A, GenBank accession No. NM_001504; GenScript) and CXCR3 transcript variant B (CXCR3B, GenBank accession No. NM_001142797; GenScript) and HEK293 cells were purchased from GenScript Biotech (Piscataway, NJ) and ATCC (Manassas, VA), respectively. Then the cDNA constructs were transfected to HEK293 cells, and the transfected cells were cultured to get enough amount for the cell-binding assay. After the CXCR3-transfected cell membranes were harvested, competitive binding assays were performed by measuring the concentration of free [^125^I]CXCL10 at different concentrations of **1** and NBI74330. Their competitive binding curves are shown in Additional file 1: Fig. S2. The IC_50_ value of **1** was measured based on the regression plot between the free radioligand and the different concentrations of **1**. The K_i_ values of **1** were calculated by the Cheng-Prusoff equation, indicating that 0.81 ± 0.02 nM and 0.31 ± 0.02 nM in CXCR3A and CXCR3B, respectively.

### PET imaging studies

[^18^F]**1** was further evaluated for reliability as a CXCR3 radiotracer using mouse models of atherosclerosis. For this purpose, we tested [^18^F]**1** in ApoE KO mice, a widely used murine model of atherosclerosis [25]. Six-week-old ApoE KO male mice (N = 6) and C57BL/6 male control mice (i.e., a background strain of ApoE KO mouse, ApoE-wildtype control; N = 6) were purchased from Jackson Laboratory (Bar Harbor, ME). The ApoE KO mice were placed on a high-fat diet over 12 weeks to accelerate atherosclerosis development. Meanwhile, C57BL/6 mice were fed a normal diet for the same period, and they did not produce atherosclerosis in the aorta. At 12 weeks, PET/computed tomography (CT) studies were performed using both mice groups. Dynamic PET scans (90 min acquisition) were performed beginning 5 min after the intravenous injection of [^18^F]**1** followed by a ~ 10 min CT scan. Usually, one week after the baseline PET/CT scan, the corresponding blocking PET/CT scan was performed. This time, **1**·HCl salt (5 mg/kg) was used as a blocking agent and intravenously injected 5 min before the radiotracer injection.

**Fig. 2:**
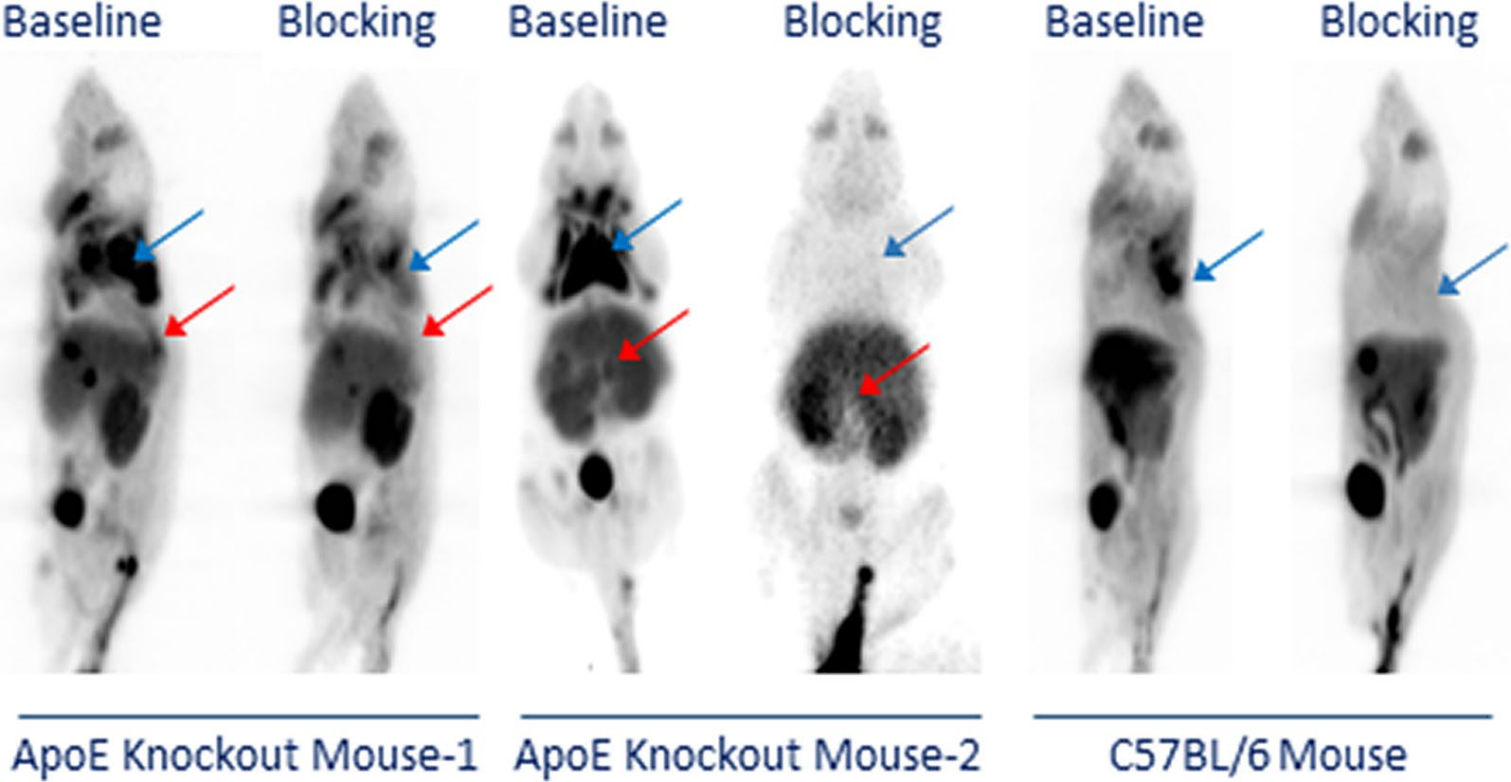
Representative CXCR3 PET images of [^18^F]1 in two ApoE KO mice and one C57BL/6 control mouse. The specific uptake of [^18^F]**1** was observed in the atherosclerotic aorta (red arrow) and BAT (blue arrow) of ApoE KO mice and the BAT of C57BL/6 control mice

Figure 2 shows the representative PET images of two ApoE KO mice and one C57BL/6 mouse, and the blocking images of the same mouse is shown next to the baseline images. The PET images of the ApoE KO mice showed high uptake of [^18^F]**1** in the atherosclerotic abdominal aorta (red arrow), brown adipose tissue (BAT, blue arrow), liver, kidney, and bladder. The PET images of the C57BL/6 mouse displayed a similar uptake except for the atherosclerotic abdominal aorta. The blocking drug appeared to reduce the tracer uptake significantly in both the BAT and atherosclerotic abdominal aorta in the ApoE KO mice. In contrast, reduced tracer uptake was observed only in the BAT region in the C57BL/6 control mice, demonstrating that [^18^F]**1** is a CXCR3-specific radiotracer. The high uptake in the liver and bladder reflects the metabolism of [^18^F]**1**. The atherosclerotic plaques were observed at multiple sites in the abdominal aorta. The skeleton-rendered PET/CT images for the baseline PET are also shown in Additional file 1: Fig. S3.

**Fig. 3:**
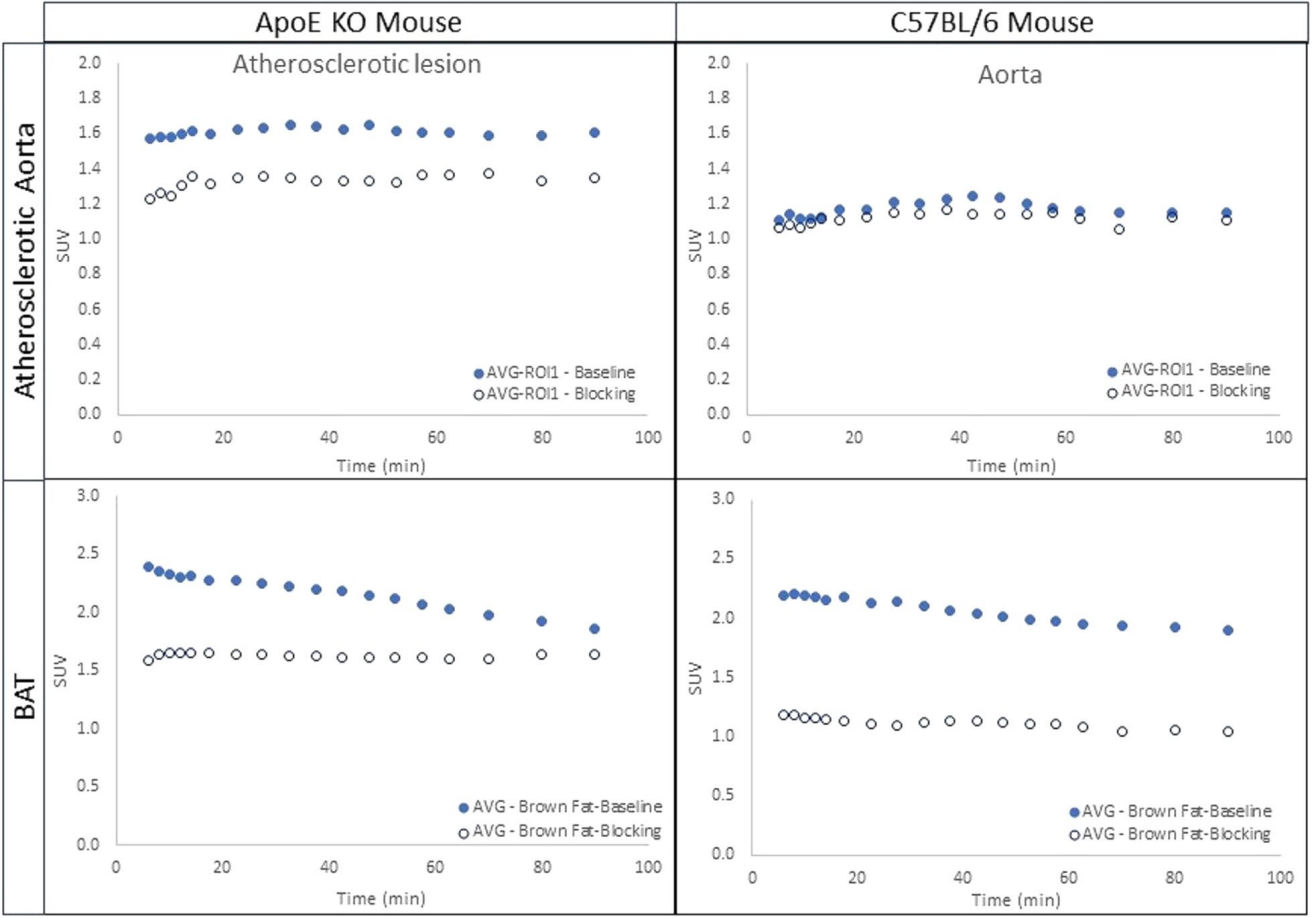
The TACs of ApoE KO mice (N = 3) and C57BL/6 control mice (N = 5). The TACs indicate the blocking effects of [^18^F]**1**, demonstrating the specificity of [^18^F]**1**

**Fig. 4:**
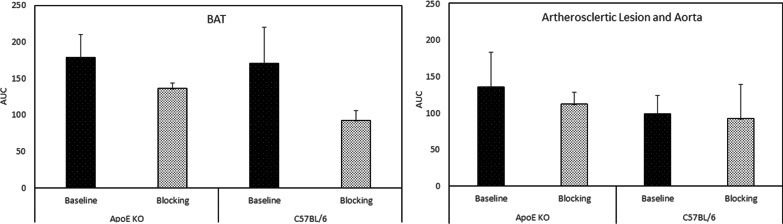
The AUCs of the BAT and atherosclerotic aorta of ApoE KO mice and the BAT and corresponding aorta of C57BL/6 mice

Figure 3 indicates the average time-activity curves (TACs) in the atherosclerotic lesion and BAT of both ApoE KO mice (N = 3) and C57BL/6 control mice (N = 5). Preadministration of **1·**HCl (5 mg/kg) in blocking studies reduced the uptake of [^18^F]**1** in the atherosclerotic abdominal aorta (standard uptake value (SUV) 1.1) in ApoE KO mice and reduced the uptake in BAT region (SUV 1.2–1.5) in both groups. A comparison of TACs from all the PET scans showed that the binding of [^18^F]**1** is specific to CXCR3, especially in these regions of interest (ROIs). The uptake levels in all the scans in ROI were retained at the more or less same level throughout the 90 min scan, indicating a slower washout of the tracer from these regions. Figure 4 displays the area under the curves (AUCs) obtained based on the TACs. The blocking effects in the BAT of ApoE KO mice and C57BL/6 control mice were 23.4% and 45.9%, and those in the atherosclerotic abdominal aorta were 16.9% and 4.7%, respectively.

### Biodistribution studies

Biodistribution studies were also performed to discover the drug distribution within normal mice. 17-week-old male and female C57BL/6 mice were purchased to match the age of the imaging study group. [^18^F]**1** was intravenously injected into these mice (N = 4/time point), then they were sacrificed at 10, 30, and 60 min after the injection. Their major organs, including the brain, blood, lung, liver, stomach, heart, spleen, kidney, muscle, bone, large intestine, small intestine, aorta, liquinal lymph nodes (LN), interscapular BAT, and tail, were harvested. Their radioactivity was counted by an automated γ-counter. The biodistribution was calculated as an average of the percent injected dose per gram tissue (%ID/g tissue) where the counted radioactivity of each organ was divided by the weight of each tissue and the total radioactivity that circulated in the mouse body at the time of the euthanasia.

**Fig. 5:**
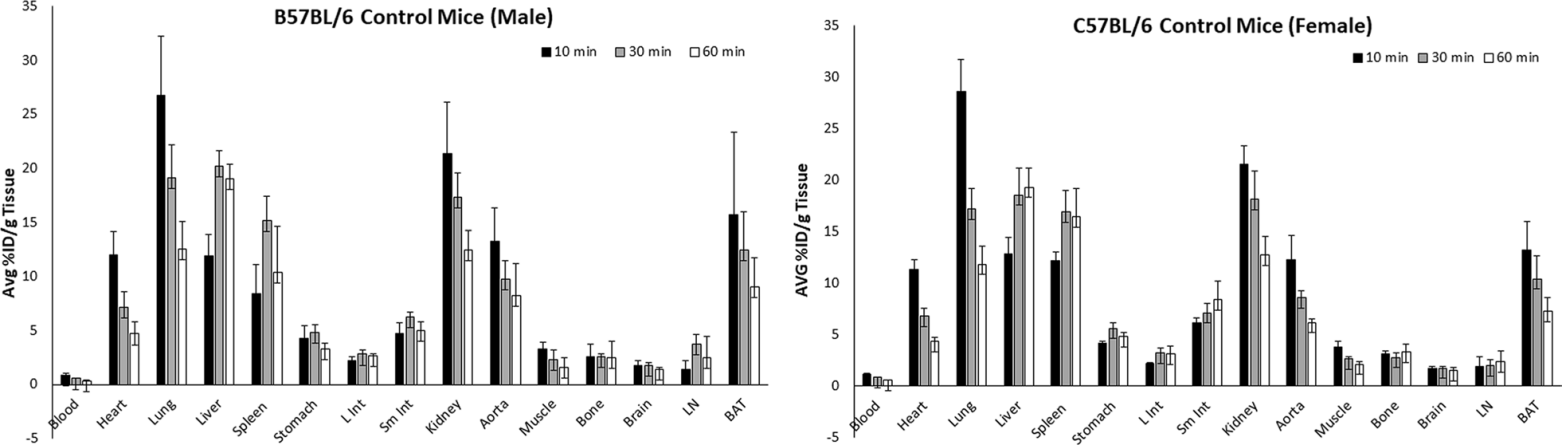
The results of biodistribution using male and female control C57BL/6 mice. The uptake of [^18^F]**1** in each organ was measured at 10, 30, and 60 min after its intravenous injection

Figure 5 shows the results of the biodistribution study. The results show that the uptake properties of [^18^F]**1** were similar between males and females. [^18^F]**1** displayed high uptake in the lung (27% ID/g) and kidney (22% ID/g) followed by the BAT (15% ID/g), aorta (13% ID/g), and heart (12% ID/g) at 10 min postinjection (p.i). At 30 min p.i., the uptake was reduced to ~ 60% and then to ~ 40% at 60 min p.i in these regions. However, the radiotracer uptake is relatively low (12% ID/g) at 10 min p.i but increased to 22% ID/g at 30 min p.i in the liver, indicating the radiotracer’s metabolism.

### Tissue analysis and immunohistochemistry (IHC) analysis

**Fig. 6:**
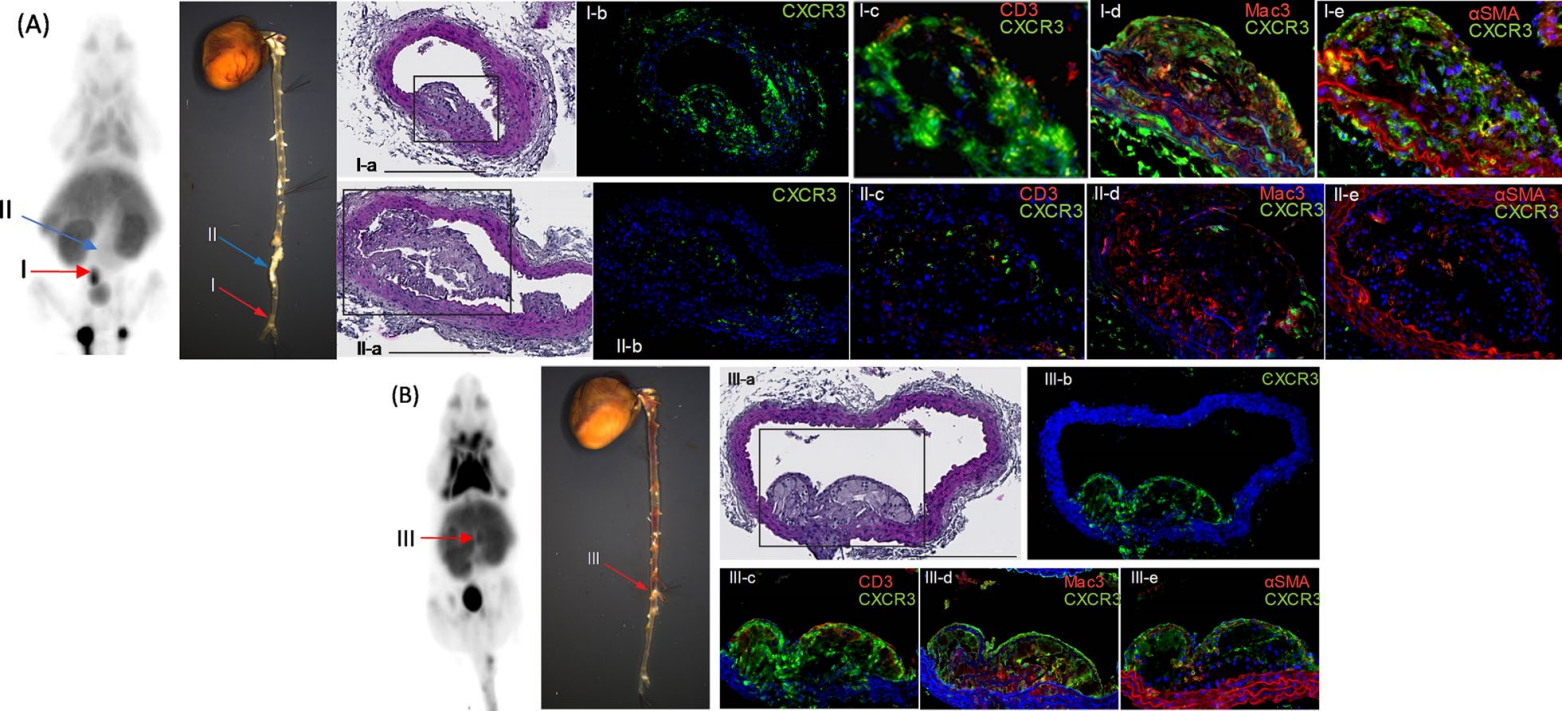
CXCR3 PET imaging using [^18^F]**1** and their corresponding cardiovascular tissues obtained from the ApoE KO mice. **A** Region I (red arrows) is a [^18^F]**1**-positive region, and the corresponding aorta tissue was tested for expression levels of CXCR3 protein, co-stained with cell-type markers. Adjacent cross sections of the region I (I-a; hematoxylin and eosin (H&E) stain) were subjected to immunofluorescent staining identifying CXCR3 (I-b–I-e; green), CD3 (I-c, red; T cell marker), Mac3 (I-d, red; macrophage marker), and αSMA (I-e, red; SMC marker), counterstained with 4′,6-diamidino-2-phenylindole (DAPI, blue; nuclei). Panels I-c–I-e are magnified views of atherosclerotic plaque indicated on I-a by a rectangle. Region II (blue arrows) has a significant atherosclerotic plaque. Nevertheless, it is a [^18^F]**1**-negative region of PET imaging. Adjacent cross sections of the region II (II-a; H&E stain) were also assessed for expression levels of CXCR3 (II-b–II-e; green), co-stained with CD3 (II-c, red), Mac3 (II-d, red), and αSMA (II-e, red) and counterstained with DAPI (blue). Panels II-c–II-e are magnified views of atherosclerotic plaque indicated on II-a by a rectangle. Scale bar = 200 µm. **B** Region III (red arrows) is a [^18^F]**1**-positive region, and the corresponding aorta tissue was tested for expression levels of CXCR3 protein, co-stained with cell-type markers. Region III also indicated [^18^F]**1**-positivity, and subsequent histologic analysis revealed a significant atherosclerotic plaque with high CXCR3 expression levels (III-b–III-e; green detection in the fluorescence images), consistent with CXCR3 expression-dependent detection by the radiotracer. Adjacent cross sections of the region III (III-a; H&E stain) were CD3 (III-c, red), Mac3 (III-d, red), and αSMA (III-e, red), counterstained with DAPI (blue). Panels III-c–III-e were magnified views of atherosclerotic plaque indicated on III-a by a rectangle. Scale bar = 200 µm

Figure 6 shows CXCR3 expression within atherosclerotic plaques, which were PET-positive (Fig. 6A, B; regions I and III on PET and en face images, indicated by red arrows) and PET-negative (Fig. 6A; region II on PET and en face images, indicated by a blue arrow). Cross sections of the regions (Fig. 6I-a, II-a, III-a) were subjected to immunofluorescence detection of CXCR3 (green), co-detected with CD3 (T cell marker), Mac3 (macrophage marker), and α-smooth muscle actin (αSMA; smooth muscle marker). The atherosclerotic plaque at Regions I and III, which were detected by PET via [^18^F]**1**, had notably higher levels of CXCR3 expression than the one in Region II (Fig. 6; compare I-b or III-b to II-b), consistent with CXCR3-specific radiotracer-dependent detection. Co-staining with cell-type markers on PET-positive plaques (Regions I and III) revealed that T cells (CD3+ , *red* on panels I-c and III-c) on the luminal surface of the plaque, macrophages (Mac3+ , *red* on panels I-d and III-d) in the core part of plaque, and smooth muscle cells (SMCs) (αSMA+ , *red* on panels I-e and III-e) underneath the endothelium and in tunica media below the plaque are highly positive for CXCR3. On the other hand, in the PET-negative plaque (Region II), there are few T cells on the luminal surface but below the endothelium, which were CXCR3-expression positive (panel II-c). Macrophages are the major cell population (panel II-d) in a deep part or core of the plaque, surrounded by SMCs (panel II-e). Interestingly, the majority of macrophages are negative for CXCR3, but a fraction of macrophages and SMCs near the perimeter of the plaque were CXCR3 expression positive. These results support CXCR3 expression-dependent detection of atherosclerotic plaques, where major cell types responsible for atherogenesis (i.e., T cells, macrophages, and SMCs) are highly expressing CXCR3.

## Discussion

We chose **1** from the reported CXCR3 ligand libraries because of its subnanomolar affinity and feasibility of labeling with fluorine-18 and decided to develop [^18^F]**1** for imaging of CXCR3 receptor. We performed a CAM study to determine the CXCR3 binding pockets and the ligand binding interactions (hydrophobic and π–π in this case) that lead to a stable receptor-ligand complex. Reference standard **1** and its precursor **9** were prepared over five steps from commercially available starting materials. Although copper or palladium-catalyzed methods are available for aromatic ^18^F-substitution using arylboronate or aryltin precursors, we presumed that benzylamines are not appropriate precursors for such labeling reactions. In addition, the occurrence of protodestannylation and protodeboronylation side reactions during ^18^F-labeling and the presence of two chlorine atoms on two different aromatic positions in **1** made us use a non-catalyzed multi-step radiosynthesis [26, 27]. The first radiosynthesis strategy that rapidly converted benzaldehyde derivative [^18^F]**15** to benzyl bromide derivative [^18^F]**17** via benzyl alcohol derivative [^18^F]**16** failed in the final step that involved the coupling of [^18^F]**17** and **9**. We presumed that the failed final step might be due to the presence of residual water and the decomposition of impure [^18^F]**17** during the reaction [22]. In addition, technical differences in synthesis setup compared to the literature method amounted to moderate conversion of [^18^F]**16** to [^18^F]**17**. Then, we tried two-step radiosynthesis that coupled [^18^F]**15** with **9** with reductive amination, which turned out to be a more efficient method [23]. The result also indicated that the acidic catalyst improved the reductive amination yield.

Cell-binding assays indicated that the K_i_ values of **1** were 0.81 ± 0.02 nM and 0.31 ± 0.02 nM in CXCR3A and CXCR3B, respectively. Therefore, **1** binds to two CXCR3 variants with the same affinity. CXCR3A mediates the chemotaxis of immune cells [24]. CXCR3A and CXCR3B co-express in the immune cells, but CXCR3A expresses almost 1000 times more abundant than CXCR3B [28]. CXCR3A and CXCR3B are also preferentially expressed in some types of cells. For example, CXCR3A is primarily expressed in human mesangial cells, while CXCR3B is preferentially expressed in microvascular endothelial cells [28]. CXCR3A and CXCR3B couple with inhibitory G (G_i_) protein and stimulatory G alpha (G_s_) protein, respectively, so their functional roles are opposite [28]. Such distinctive functional differences are prominent in cancer biology, where CXCR3A promotes the migration of tumor cells and angiogenesis, but CXCR3B inhibits such metastatic properties and promotes apoptosis in many cancers [29]. Overall, the PET images using [^18^F]**1** might indicate all CXCR3 variants depending on their expression levels. When the PET images of [^18^F]**1** indicate high inflammatory regions, it is expected to represent CXCR3A because of its predominant expression.

The baseline and blocking PET studies and IHC studies demonstrated that [^18^F]**1** was a specific CXCR3 radiotracer to atherosclerotic lesions. The biodistribution and PET imaging studies also indicated that the uptake of [^18^F]**1** in the atherosclerotic aorta was obvious in the ApoE KO mice, despite that its background uptake was high in the control C57BL/6 mice. The fatality of atherosclerosis comes from its lesions in the coronary and carotid arteries, which cause myocardial infarction, angina, and stroke in humans. Therefore, further studies using other animal models that develop atherosclerotic plaques in those regions are required to determine if this radiotracer can identify atherosclerotic lesions in the heart and other critical regions. We also need further studies to evaluate the sensitivity of these compounds to identify inflammatory lesions in the lung, liver, and intestine since the uptake of [^18^F]**1** was very high in normal C57BL/6 mice in biodistribution studies. This is important because various inflammatory diseases such as chronic obstructive pulmonary disease, hepatic cirrhosis, and Crohn’s disease, which feature inflammation mechanisms in their pathologies, develop in those regions, respectively.

The PET images also indicated that the uptake of [^18^F]**1** was specific in the BAT. It has been reported that CXCR3 mediates T-lymphocyte migration to the adipose tissue under chronic inflammatory conditions, in particular, induced by obesity [30, 31]. In addition, a high-fat diet in rodents was shown to induce CXCR3 expression in the adipose tissue as a result of increased inflammation [32]. In the current study, the diet schedule may have induced chronic inflammation in the adipose tissue of both the ApoE KO mice and C57BL/6 control mice, resulting in high uptake of [^18^F]**1** in the BAT region.

The IHC analysis using the aorta tissues of ApoE KO mice that were imaged for PET indicated that not all atherosclerotic lesions were identified by [^18^F]**1**. This result was also related to the sensitivity of [^18^F]**1**. Further studies are required to test the sensitivity of this radiotracer. Our study also demonstrated that [^18^F]**1** detects specific atherosclerotic lesions where CXCR3 expression is high, consistent with the high affinity of [^18^F]**1** to CXCR3. Co-staining with cell-type markers indicated that not only T cells but also macrophages and SMCs were highly expressing CXCR3 in the plaque. In fact, previous reports describe that all these cell types express CXCR3, potentially promoting atherosclerosis [8, 33]. Meanwhile, atherosclerotic plaques, which [^18^F]**1** did not indicate, are suggested to have low levels of CXCR3 expression, whilst such the plaques still indicate inflammatory phenotype with a major existence of macrophages (Fig. 6, panel II-d), which also agrees with the preclinical PET imaging studies in the literature that tracked the infiltration of macrophages that express CXCR4 and somatostatin-2 receptors [34, 35]. CXCR3 expression levels in plaques are disease progression-dependent, so [^18^F]**1**–positive indication could potentially reflect the status. It has been shown that CXCR3-expressing T cells accumulate early in atheroma formation and persist as the lesion grows and ruptures [36]. Intriguingly, lower levels of CXCR3 expressions were found in the peripheral cells of patients with acute coronary syndrome, which may reflect a sequestering of circulating CXCR3 positive cells to the advanced atheroma causing infarction [33, 37]. Macrophages, as well as SMCs, are highly plastic, gaining a broad spectrum of their activation and differentiation statuses under the influence of the pro-atherogenic milieu of the lesion, and CXCR3 mediates proinflammatory and pro-atherogenic effects of its cognate ligands in macrophages and SMCs [36, 38]. CXCR3 is a receptor for CXCL9, CXCL10, and CXCL11, which are IFN-γ inducible and thus associated with inflammatory disorders, including atherosclerosis [6]. It has been suggested that CXCR3 has integral roles in the recruitment of proinflammatory cells during plaque formation, thereby promoting atherogenesis [9], however its potential role in the progression of plaque leading to an advanced lesion (i.e., a plaque to cause an acute thromboembolic event or Type V and VI lesions [39]) remains obscure. Therefore, an interesting question is raised at this point whether the positive detection by the radiotracer (thus high levels of CXCR3 expression) could reflect any pathologic response by plaque cells, thus a pathophysiologic status of the plaque. Further studies are needed to clarify how CXCR3 expression levels transition as an atheroma evolves from the initial fatty streak to an advanced lesion so that the clinical implications of CXCR3-positive plaques, thus the PET detection, can be evaluated.

## Conclusions

The target compound **1**, a derivative of VUF11211, was identified as a lead compound for PET radiotracer for CXCR3 chemokine receptors as a result of a literature search and CAM study. After the target compound **1** and its precursor compound **9** were prepared by multi-step organic synthesis, cell-binding assays confirmed its binding affinity. [^18^F]**1** was prepared by an F-18 incorporation followed by reductive amination, a one-pot and two-step radiosynthesis. [^18^F]**1** was validated as a CXCR3 PET radiotracer using mouse models of atherosclerosis. The baseline scan and blocking PET scan, which pre-administered **1**.HCl salt intravenously, confirmed that [^18^F]**1** is a CXCR3-specific radiotracer and indicated atherosclerotic plaques in the abdominal aorta of ApoE KO mice. The IHC studies using the atherosclerotic abdominal aorta also confirmed that the radiotracer indicated CXCR3-abundant aorta regions. However, the radiotracer did not detect some atherosclerotic plaques, which have a similar size to detectable plaques. IHC revealed that these undetected plaques have low levels of CXCR3 expression, indicating a lack of correlation between the plaque size and CXCR3 expression levels and the nonspecificity of CXCR3 expression by various types of immune cells and SMCs. Further analysis is required to correlate the CXCR3 PET imaging with the plaque progression. In addition, the biodistribution studies using control C57BL/6 mice showed high background in the lungs, heart, aorta, liver, and intestine. The new radiotracer successfully indicated the atherosclerotic abdominal aorta in the ApoE KO mice despite the high background uptake in the regions of C57BL/6 control mice. Therefore, further studies are also required to demonstrate the versatility of [^18^F]**1** in detecting inflammatory lesions in the coronary artery, liver, lungs, and intestine. In conclusion, [^18^F]**1** is the first promising F-18 radiotracer for CXCR3, and its properties, such as CXCR3 sensitivity, have to be improved with further development.

## Methods

### General information

All the reactions were carried out in oven-dried glassware under an atmosphere of nitrogen or argon. All commercially obtained reactants and reagents were used as such without any further purification. Unless specified, all chemicals were purchased from MilliporeSigma (Burlington, MA). All purification solvents such as hexane, ethyl acetate (EtOAc), dichloromethane (DCM), and MeOH, and all anhydrous solvents such as dioxane, DCM, and DMF were purchased from Fisher Scientific (Waltham, MA). For lower temperature reactions, an ice bath (0°C) and an acetone-dry ice bath (-78 °C) were used. Reactions were monitored by TLC using silica gel-coated aluminum plates with F-254 indicator. ^1^H (500 MHz), ^13^C (126 MHz), ^19^F (476 MHz) nuclear magnetic resonance (NMR) spectra were recorded on Bruker Avance III 500 MHz spectrometer. Electrospray (ESI) and Atmospheric Pressure Chemical Ionization (APCI) mass spectra (MS) and high-resolution mass spectra (HRMS) were obtained using a Thermo LCQ Deca XP Ion Trap Mass Spectrometer and a Bruker Impact II Q-TOF Mass Spectrometer, respectively. The NMR spectra and MS spectra can be found in Additional file 1: Fig. S4.

## Organic synthesis

### Methyl (S)-6-(4-(tert-butoxycarbonyl)-3-ethylpiperazin-1-yl)-5-chloronicotinate (4)

To the solution of methyl 5,6-dichloronicotinate (**2**, 2.200 g, 10.680 mmol) in anhydrous DMF (25 mL) was added *tert*-butyl (S)-2-ethylpiperazine-1-carboxylate (**3**, 2.500 g, 11.747 mmol) and DIPEA (9.500 mL, 53.398 mmol) under nitrogen. The reaction contents were then heated to 90°C and stirred for 18 h. Following this time, reaction was cooled, quenched with water (40 mL) and EtOAc (40 mL), and added 1 M sodium bicarbonate (NaHCO_3_) solution (40 mL). The aqueous layer was extracted with EtOAc (3 × 30 mL), and the combined organic layers were washed with brine (50 mL) and dried over anhydrous Na_2_SO_4_. The crude was concentrated and purified by column chromatography (0–20% EtOAc in Hexanes). **4** was obtained as a pale-yellow gummy solid. R_f_: 0.4 (20% EtOAc in hexane). Yield: 3.530 g, 9.212 mmol, 86.3%. ^1^H NMR (500 MHz, chloroform-d (CDCl_3_)) δ 8.71 (d, J = 2.0 Hz, 1H), 8.11 (d, J = 2.0 Hz, 1H), 4.14–3.96 (m, 4H), 3.89 (s, 3H), 3.19 (q, J = 10.4, 8.0 Hz, 1H), 3.08 (dd, J = 12.9, 3.6 Hz, 1H), 2.98–2.88 (m, 1H), 1.79 (dt, J = 15.0, 7.6 Hz, 1H), 1.70–1.57 (m, 1H), 1.47 (s, 9H), 0.87 (t, J = 7.5 Hz, 3H). ^13^C NMR (126 MHz, CDCl3) δ 171.14, 165.11, 160.39, 154.93, 147.78, 139.93, 119.78, 119.53, 79.78, 60.39, 52.16, 50.71, 48.54, 28.44, 14.20, 14.12, 10.71. HR-ESI–MS [M + Na]^+^ C_18_H_26_ClN_3_O_4_, calcd 406.15095, found [M + Na]^+^ 406.14997.Additional file 1: Fig. S4(a).

### Methyl (S)-5-chloro-6-(3-ethylpiperazin-1-yl)nicotinate (5)

To the solution of **4** (2.980 g, 7.763 mmol) in dry DCM (10 mL) at 0°C was added dropwise TFA (6 mL), warmed to room temperature (RT), and stirred for 90 min. Following this time, the crude was basified using 5.9 g K_2_CO_3_ in 100 mL water (pH > 7) and extracted with DCM (3 × 70 mL). Combined organic layers are dried over anhydrous Na_2_SO_4_ and concentrated under reduced pressure to obtain **5** as a white solid and used as such in next reaction. R_f_ 0.5 (10% MeOH in DCM). Yield: 2.160 g, 7.609 mmol, 98.2%. ^1^H NMR (500 MHz, CDCl_3_) δ 8.73 (d, J = 2.0 Hz, 1H), 8.11 (d, J = 2.0 Hz, 1H), 4.34–3.95 (m, 2H), 3.89 (s, 3H), 3.44–2.92 (m, 3H), 2.78 (ddt, J = 9.4, 6.5, 3.3 Hz, 1H), 2.65 (dd, J = 12.4, 10.1 Hz, 1H), 1.64–1.41 (m, 2H), 0.99 (t, J = 7.5 Hz, 3H). ^13^C NMR (126 MHz, CDCl_3_) δ 165.16, 160.02, 147.73, 139.84, 119.80, 119.12, 56.55, 54.29, 52.08, 49.27, 45.72, 26.87, 10.17. HR-ESI–MS [M + H]^+^ C_13_H_18_ClN_3_O_2_, calcd 284.10875, found 284.11518. (Additional file 1: Fig. S4(b)).

### Methyl (S)-6-(4-(1-(tert-butoxycarbonyl)piperidin-4-yl)-3-ethylpiperazin-1-yl)-5-chloronicotinate (7)

To the **5** (1.110 g, 3.886 mmol) in 1,2-dichloroethane (DCE, 10 mL) was added anhydrous magnesium sulfate (MgSO_4_, 0.935 g, 7.772 mmol) and slowly 1-Boc-4-piperidinone (**6**, 0.770 g, 3.886 mmol) dissolved in 3 mL DCE and stirred for 30 min at RT. Following this time, Sodium triacetoxyborohydride (1.640 g, 7.772 mmol) was added in 4 portions over 15 min and the slurry was stirred for 24 h. Following this time, the reaction was quenched with NaHCO_3_ (3 g in 100 mL water) and extracted with DCM (3 × 40 mL). Combined organic layers were dried over Na_2_SO_4_ and the crude was purified using column chromatography. 0–50% EtOAc in hexane used initially to collect **7** as a gummy solid. Then 0–10% MeOH in DCM was used to elute the unreacted starting material (0.460 g). Yield: 0.930 g, 1.996 mmol, 54%. ^1^H NMR (500 MHz, CDCl_3_) δ 8.72 (d, J = 2.0 Hz, 1H), 8.10 (d, J = 2.0 Hz, 1H), 4.15 (d, J = 18.5 Hz, 3H), 3.89 (s, 3H), 3.85–3.74 (m, 2H), 3.33 (ddd, J = 12.2, 8.9, 2.8 Hz, 1H), 3.23 (dd, J = 12.5, 7.8 Hz, 1H), 2.85 (ddd, J = 11.4, 5.8, 3.2 Hz, 2H), 2.74 (ddt, J = 8.2, 5.0, 3.0 Hz, 1H), 2.63 (ddd, J = 11.5, 8.8, 3.0 Hz, 1H), 1.78–1.54 (m, 6H), 1.46 (s, 9H), 0.94 (t, J = 7.5 Hz, 3H). ^13^C NMR (126 MHz, CDCl_3_) δ 165.21, 159.86, 154.69, 147.77, 139.83, 119.35, 118.87, 79.44, 57.09, 55.63, 52.07, 51.74, 48.81, 44.35, 28.43, 20.68, 14.18, 10.12. HR-ESI–MS [M + H]^+^ C_23_H_35_ClN_4_O_4_, calcd 467.24251 found 467.24061. (Additional file 1: Fig. S4(c)).

### Tert-Butyl (S)-4-(4-(3-chloro-5-(1,3,4-oxadiazol-2-yl)pyridin-2-yl)-2-ethylpiperazin-1-yl)piperidine-1-carboxylate (8)

To the** 7** (0.610 g, 1.308 mmol) in 5 mL EtOH was added 64% hydrazine monohydrate (0.500 mL, 10.464 mmol) and stirred at 50°C for 12 h. Following this time, the volatile components were evaporated and kept under a high vacuum for 2 h to obtain a colourless solid that was used as such in the next step. The hydrazide intermediate was dissolved in chloroform (6 mL) and triethyl orthoformate (4 mL) and refluxed (65°C) for 16 h. Following this time, pyridine-p-toluene sulfonate (0.700 g, 2.616 mmol) was added and refluxed (65°C) for another 4 h. The reaction was basified using 0.5 g sodium carbonate in 100 mL water and extracted with DCM (3 × 35 mL). Organic layers were dried over anhydrous Na_2_SO_4_ and purified using 0–5% MeOH in DCM to obtain **8** as a white solid. Yield: 0.425 g, 0.892 mmol, 68% over 2 steps. ^1^H NMR (600 MHz, CDCl_3_) δ 8.80 (d, *J* = 2.1 Hz, 1H), 8.46 (s, 1H), 8.22 (d, *J* = 2.1 Hz, 1H), 4.28–4.12 (m, 2H), 3.86–3.77 (m, 2H), 3.38 (ddd, *J* = 12.2, 8.8, 2.8 Hz, 1H), 3.27 (dd, *J* = 12.5, 7.8 Hz, 1H), 2.90 (dtt, *J* = 11.4, 5.6, 3.1 Hz, 2H), 2.78 (tt, *J* = 8.0, 3.0 Hz, 2H), 2.67 (ddd, *J* = 11.6, 8.8, 3.0 Hz, 2H), 1.76–1.70 (m, 3H), 1.70–1.57 (m, 5H), 1.49 (s, 9H), 0.98 (t, *J* = 7.4 Hz, 3H). ^13^C NMR (126 MHz, CDCl_3_) δ 162.47, 159.46, 154.83, 152.49, 144.67, 137.17, 120.50, 112.89, 79.60, 57.23, 55.80, 53.56, 51.94, 49.00, 44.48, 30.79, 28.58, 25.33, 20.83, 10.28. HR-ESI–MS [M + H]^+^ C_23_H_33_ClN_6_O_3_, calcd 477.23809, found 477.23714. Additional file 1: Fig. S4(d).

### (S)-2-(5-Chloro-6-(3-ethyl-4-(piperidin-4-yl)piperazin-1-yl)pyridin-3-yl)-1,3,4-oxadiazole (9)

To the **8** (0.130 g, 0.273 mmol) dissolved in DCM (3 mL) at 0°C was slowly added TFA (1.5 mL). The reaction was then warmed to RT and stirred for 2 h. Following this time, the reaction was quenched at 0°C with 1.9 g NaHCO_3_ in 45 mL water (pH between 7–8) and extracted with DCM (3 × 15 mL). The organic layers were washed with 10 mL brine. The combined organic layers were then dried and concentrated to obtain **9** as a semi-white solid. Yield: 0.094 g, 0.250 mmol, 91.2%.

^1^H NMR (500 MHz, CDCl_3_) δ 8.78 (d, *J* = 2.1 Hz, 1H), 8.46 (s, 1H), 8.20 (d, *J* = 2.1 Hz, 1H), 3.83–3.70 (m, 2H), 3.52–3.41 (m, 2H), 3.38 (ddd, *J* = 12.1, 8.4, 2.7 Hz, 1H), 3.30 (dd, *J* = 12.7, 7.5 Hz, 1H), 3.01–2.80 (m, 4H), 2.75 (pd, *J* = 8.3, 5.4, 3.0 Hz, 1H), 2.67 (ddd, *J* = 11.3, 8.4, 2.9 Hz, 1H), 2.05 (qd, *J* = 12.7, 11.2, 7.3 Hz, 1H), 1.96–1.80 (m, 3H), 1.67–1.52 (m, 2H), 0.95 (t, *J* = 7.4 Hz, 3H). ^13^C NMR (126 MHz, CDCl_3_) δ 162.30, 159.32, 152.36, 144.51, 137.04, 120.49, 112.93, 56.93, 53.40, 51.58, 48.78, 44.21, 44.13, 43.82, 28.36, 23.59, 20.40, 10.14. HR-ESI–MS [M + H]^+^ C_18_H_25_ClN_6_O, calcd 377.18566, found 377.18459. (Additional file 1: Fig. S4(e)).

### *N*-(4-Chloro-2-fluorobenzyl)-4-piperidinone (12)

To a mixture solution of 4-piperidinone hydrochloride monohydrate (**10**, 1.000 g, 6.510 mmol) and K_2_CO_3_ (2.249 g, 16.27 mmol) in MeCN (13 mL) was added 4-chloro-2-fluorobenzyl bromide (**11**, 1.06 ml, 7.812 mmol) and refluxed overnight. The resulting solution was quenched with sat. ammonium chloride and extracted three times with EtOAc. The organic parts were combined, dried over anhydrous Na_2_SO_4_, filtered, and evaporated. The residue was purified by flash chromatography using gradient solvent by increasing from 5 to 30% EtOAc in hexane for 30 min to give **12** as a yellow oil. 1.251 g, 5.176 mmol, 79.5%.

^1^H NMR (500 MHz, CDCl_3_) δ 7.37 (t, *J* = 8.0 Hz, 1H), 7.13 (dd, 1H,* J* = 8.0, 2.0 Hz, 1H), 7.09 (dd, *J* = 9.5, 2.0 Hz, 1H), 3.66 (d,* J* = 1.0 Hz, 2H), 2.77 (t, *J* = 6.0 Hz, 4H), 2.46 (t,* J* = 6.3 Hz, 4H). ^13^C NMR (126 MHz, CDCl_3_) δ 208.87, 161.17 (d, *J* = 249 Hz), 134.02 (d, *J* = 11.25 Hz), 132.06 (d, *J* = 2.5 Hz), 124.63 (d, *J* = 3.8 Hz), 123.58 (d, *J* = 15 Hz), 116.34 (d, *J* = 25 Hz), 54.00 (d, *J* = 1.3 Hz), 52.88, 41.35. HR-ESI–MS [M]^+^ C_12_H_13_FClNO, calcd 241.07, found [M + H]^+^ 242.39. (Additional file 1: Fig. S4(f)).

### Methyl (S)-5-chloro-6-(4-(1-(4-chloro-2-fluorobenzyl)piperidin-4-yl)-3-ethylpiperazin-1-yl)nicotinate (13)

To the **5** (0.330 g, 1.166 mmol) in 5 mL DCE was added MgSO_4_ (0.280 g, 2.332 mmol) and slowly 1-(4-chloro-2-fluorobezyl)piperidin-4-one (**12**, 0.420 g, 1.749 mmol) dissolved in 3 mL DCE and stirred for 60 min at RT. Following this time, sodium triacetoxyborohydride (0.500 g, 1.749 mmol) was added in 4 portions, and the slurry was stirred for 60 h. The reaction was quenched with NaHCO_3_ solution (1.2 g in 70 mL water) and extracted with DCM (3 × 40 mL). Combined organic layers were dried over anhydrous Na_2_SO_4_, and the crude was purified using column chromatography. 0–5% MeOH in DCM was used to elute **13** as a yellow gummy solid. Yield: 0.310 g, 0.610 mmol, 53%. ^1^H NMR (500 MHz, CDCl_3_) δ 8.72 (d, J = 2.0 Hz, 1H), 8.09 (d, J = 2.0 Hz, 1H), 7.33 (td, J = 8.1, 2.8 Hz, 1H), 7.13–7.09 (m, 1H), 7.06 (dt, J = 9.6, 2.2 Hz, 1H), 3.89 (s, 3H), 3.87–3.78 (m, 2H), 3.69 (tt, J = 9.2, 4.4 Hz, 1H), 3.54 (dd, J = 4.3, 1.5 Hz, 4H), 3.30 (ddd, J = 12.4, 9.2, 2.8 Hz, 1H), 3.17 (dd, J = 12.5, 8.1 Hz, 1H), 2.92 (dddt, J = 25.1, 11.4, 5.0, 2.7 Hz, 3H), 2.81–2.66 (m, 4H), 2.63 (ddd, J = 11.7, 9.2, 3.0 Hz, 2H), 2.19 (ddd, J = 12.5, 10.0, 2.9 Hz, 2H), 2.09 (td, J = 11.5, 2.6 Hz, 1H), 2.05–1.97 (m, 2H), 1.93–1.84 (m, 1H), 1.68–1.45 (m, 2H), 0.92 (t, J = 7.4 Hz, 3H). ^13^C NMR (126 MHz, CDCl_3_) δ 165.23, 162.03, 160.05, 159.84, 147.77, 139.82, 133.38 (d, J = 10.8 Hz), 132.13 (dd, J = 8.8, 5.5 Hz), 124.25 (t, J = 3.1 Hz), 123.79 (d, J = 11.7 Hz), 119.03 (d, J = 63.5 Hz), 115.96 (d, J = 26.0 Hz), 57.24, 55.47, 54.58 (d, J = 10.0 Hz), 53.31, 52.87, 52.06, 51.84, 50.70, 48.83, 44.46, 34.46, 30.59, 24.83, 20.97 (d, J = 14.9 Hz), 14.18. HR-ESI–MS [M + H]^+^ C_25_H_31_Cl_2_FN_4_O_2_, calcd 509.18863, found 509.18700. (Additional file 1: Fig. S4(g)).

### (S)-2-(5-chloro-6-(4-(1-(4-chloro-2-fluorobenzyl)piperidin-4-yl)-3-ethylpiperazin-1-yl)pyridin-3-yl)-1,3,4-oxadiazole (1)

A similar procedure to that of **8** was repeated with **13** (0.200 g, 0.393 mmol) to prepare the reference standard **1**. Yield: 0.140 g, 0.270 mmol, 69% over 2 steps. ^1^H NMR (500 MHz, CDCl_3_) δ 8.78 (d, *J* = 2.0 Hz, 1H), 8.46 (s, 1H), 8.20 (d, *J* = 2.1 Hz, 1H), 7.34 (t, *J* = 8.0 Hz, 1H), 7.12 (dd, *J* = 8.2, 2.1 Hz, 1H), 7.07 (dd, *J* = 9.6, 2.1 Hz, 1H), 3.86 (t, *J* = 16.3 Hz, 2H), 3.56 (s, 2H), 3.21 (s, 1H), 2.96 (q, *J* = 15.1, 12.0 Hz, 3H), 2.80–2.73 (m, 2H), 2.70–2.65 (m, 1H), 2.16–1.99 (m, 2H), 1.91–1.49 (m, 7H), 0.95 (t, *J* = 7.5 Hz, 3H). ^13^C NMR (126 MHz, CDCl_3_) δ 162.33, 162.04, 160.05, 159.26, 152.37, 144.53, 137.05, 132.27, 124.33 (d, *J* = 7.5 Hz), 120.39, 116.01 (d, *J* = 25.9 Hz), 112.78, 54.56, 52.99 (d, *J* = 57.0 Hz), 48.73, 44.51, 34.65, 31.59, 30.38, 24.65, 20.88, 10.13. ^19^F NMR (564 MHz, CDCl_3_) δ -115.30. HR-ESI–MS [M + H]^+^ C_25_H_29_Cl_2_FN_6_O, 519.18422, found 519.18220. (Additional file 1: Fig. S4(h)).

## Radiochemistry

### Radiosynthesis

The radiosynthesis was carried out in a semi-automatic fashion where the reagent additions and purification transfers are controlled manually from outside the shielded hood. A PETrace series 800 Medical Cyclotron (GE Healthcare, Chicago, IL) produced aqueous [^18^F]/H_2_^18^O (20–450 mCi) by ^18^O[p, n]^18^F nuclear reaction was trapped on QMA Light carbonate cartridge (Waters Corp., Milford, MA) pretreated with 10 mL of water. After the trapped radioactive fluoride was washed with 5 mL of water, it was transferred to 10 mL conical-shaped vial using 0.8 mL of K_2_CO_3_: Kryptofix222 (25 mg: 75 mg in 8 mL MeCN and 2 mL water). The residual water was azeotropically dried with MeCN (3 × 1 mL) at 100°C under an argon stream for over 15 min. The dried [^18^F]KF-Kryptofix222 complex was cooled, and to which was added 4 mg of 4-chloro-2-nitrobenzaldehyde (**14**) dissolved in 0.5 mL DMSO under an argon atmosphere. The reaction was maintained at 140°C for 15 min and cooled in RT while the oil bath cooled to 110°C. Precursor **9** (5 mg), NaBH_3_CN (11 ± 1 mg), and AcOH (10 μL) dissolved in DMSO (0.5 mL) were transferred to the radiolabelled intermediate and heated at 110°C for 20 min. The reaction was quenched with 5 mL water mixed with 1 mL of K_2_CO_3_ (1 M) and passed through a C_18_ Sep-Pak cartridge (400 mg, Waters Corp.) pretreated with 1 mL MeCN followed by 5 mL water. The cartridge was rinsed further with 6 mL water. The trapped radiolabeled compounds were eluted with 2 mL MeCN mixed with 0.5 mL DMSO. This is followed by the cartridge wash with 2.5 mL water (0.1% NEt_3_) to make the total volume of 5 mL. This solution was injected into a semi-preparative HPLC system for purification of the radiotracer that consisted of Shimadzu LC-20A pump, a Knauer K200 UV detector, and Bioscan γ-flow detector with a Phenomenex Gemini C_18_ Semi-preparative column (5 µm, 250 × 10 mm) eluting with an isocratic mobile phase composed of 65% MeCN, 5% MeOH, 30% water, and 0.1% NEt_3_ at a flow rate of 5 mL/min. The desired [^18^F]**1** was collected between 16 and 17 min into a 50 mL collection flask containing 40 mL of water. The collected HPLC fractions were passed through a C_18_ Sep-Pak cartridge pretreated with 1 mL MeCN followed by 5 mL water into the waste. The [^18^F]**1** trapped in the C_18_ Sep-Pak cartridge was then eluted using 1 mL EtOH and diluted with 9 mL of 0.9% sterile saline containing 0.5% sodium ascorbate. (Additional file 1: Fig. S1(a)).

### Quality control

The RCY of [^18^F]**1** was monitored by analytical HPLC using Phenomenex Gemini C_18_ column (250 × 4.6 mm, 5 µm) with a mobile phase consisting of 65% MeCN, 5% MeOH, 30% water, 0.1% NEt_3_ at a flow rate of 1 mL/min in an isocratic elution mode. The synthesis afforded [^18^F]**1** in decay-corrected RCY of 13 ± 2% (*n* = 10) at the EOS, > 99% RCP in a total synthesis time of 100 min. (Additional file 1: Fig. S1(b)).

### Specific activity

Specific activity was measured during the quality control of the final product using the analytical HPLC. A standard calibration curve was set up with five different concentrations of the standard solution of nonradioactive **1** by injecting them into the same analytical HPLC conditions. After the radioactivity of the final radioactive product was measured by a dose calibrator, it was injected into the analytical HPLC. The UV area of the injected product aliquot was converted into the corresponding mass. Specific activity was calculated by dividing the measured radioactivity that decay-corrected to the EOS by the molar mass of **1**. The measured specific activity was 44.4 ± 3.7 GBq/µmol at the EOS (*n* = 6).

### Computer-aided modeling

The structure of CXCR3 was generated by homology modeling using the crystal structure of the CXCR4 chemokine receptor in a complex with small-molecule antagonist IT1t resolved at 2.5 Å (PDB file 3ODU) as a template [40]. The CXCR3 sequence was aligned with the CXCR4 sequence in PDB file 3ODU using default parameters of Maestro graphical user interface of Schrodinger Suite (Schrodinger LLC, NY). The homology model was generated by the ‘Prime’ program of the Schrodinger Suite. During homology modeling, compound IT1t was removed. The final structure was subjected to restricted minimization (1,000 iterations) by ‘Impact’ module of Schrödinger Suite with OPLS_2005 force field. The resultant structure was used to identify suitable ligand binding pockets using (i) SiteMap (Schrödinger Suite, NY), and (ii) SiteID (Certara, Tripos Associates, St. Louis, MO) programs. The spatial structure of **1** was generated using the ChemSketch program (Advanced Chemistry Development, Toronto, Canada). The structure was subject to ‘LigPrep’ utility of Schrodinger Suite to generate the molecules suitable for the docking. The docking of **1** was conducted with ‘Glide’ (Schrodinger Suite) with XP (extra precision) option. The docked pose with the greatest docking score was selected to generate the figure using PyMol (Schrodinger LLC., NY).

## Cell-binding assays

### Molecular cloning and transfections

HEK293 cells were grown at 37°C in Dulbecco's Modified Eagle (DMEM) media (Corning Inc., Corning, NY) supplemented with 10% fetal bovine serum (FBS, Corning Inc.) and 1% penicillin/streptomycin/amphotericin B (Corning Inc.). cDNA constructs containing CXCR3A (GenBank accession # NM_001504; GenScript) and CXCR3B (GenBank accession # NM_001142797; GenScript) were purchased from GenScript. Prior to transfection, HEK293 cells were seeded at the appropriate density to ensure 75% cell confluence within 24 h. Transfection of the cDNA constructs was performed after 24 h using X-tremeGENE Transfection Reagent (Roche Holding AG, Basel, Switzerland) according to the manufacturer’s protocol using a 3:1 transfection reagent to cDNA ratio as previously described [41]. Following 48 h of transfection, the media was changed to complete media containing the selection agent G418 (400 µg/mL, Corning Inc.). The cells were maintained in G418 containing media to generate stable cell lines, which were used in subsequent experiments.

### Membrane preparation

Membrane preparations were prepared from CXCR3A and CXCR3B expressing HEK293 cells as previously described. In brief, the transfected cells were lysed in ice-cold lysis buffer (25 mM Tris, pH 7.4, 5 mM EDTA, 1 µg/mL aprotinin, 1 µg/mL leupeptin) and centrifuged at 1,000 × g for 5 min at 4°C. The supernatant was centrifuged at 30,000 × g, and the crude membrane pellet was resuspended in lysis buffer containing 10% glycerol and stored at -80°C until use.

### Radioligand binding

Radioligand binding was performed using crude HEK293 membranes overexpressing CXCR3A or CXCR3B as previously described [42]. Competitive radioligand binding was performed using a single concentration (80 pM) of ^125^I-CXCL10 with increasing concentrations of the CXCR3 antagonists, NBI74330 and **1**, in a 250 µL total volume of binding buffer (75 mM Tris pH 7.4, 2 mM EDTA, 12.5 mM MgCl_2_, 1 µg/mL aprotinin, 1 µg/mL leupeptin). Specific radioactive counts were plotted as a function of the competitive receptor antagonist concentration, and nonlinear regression analysis was used to determine the concentration of the receptor antagonist that reduced specific ^125^I-CXCL10 binding by 50% (IC_50_). The K_i_ values of each competitive antagonist for specific ^125^I-CXCL10 binding sites were calculated using the method of Cheng and Prusoff, as previously described [42]. The regression plots of the cell-binding assays are found in Additional file 1: Fig. S2.

## Animal studies

All animal studies were performed at the VA Biomolecular Imaging Center (VA BIC) at the Harry S. Truman Memorial Veterans’ Hospital (Columbia, MO). Six-week-old ApoE KO male mice and C57BL/6 control male mice were purchased from Jackson Laboratory. All mice were housed in the controlled vivarium during the studies. All ApoE KO mice were fed with high-fat chow that contained 42% fat, 34% of that contained unsaturated fat (TD.88137, Envigo, Indianapolis, IN), one week after the arrival and continued the feeding until the end of the study. The first PET/CT scans were performed 12 weeks after initiating the high-fat diet, and the second PET/CT scans that used **1**·HCl as a blocking agent were performed at least one week after the first PET/CT scans. Meanwhile, C57BL/6 control mice were fed with normal chow over the same schedule to match with ApoE KO mice.

### PET imaging

Maximum intensity micro-PET images were obtained on a Siemens INVEON small-animal PET/CT scanner (Siemens Medical Solutions USA, Inc., Malvern, PA). The unit has a gantry diameter of 21 cm, a transverse field of view (FOV) of 12.8 cm, and an axial length of 11.6 cm. The scanner operated in a 90 min dynamic, three-dimensional (3D) volume imaging acquisition mode. The mice were laser aligned at the center of the scanner FOV for subsequent imaging. Mice were administered 1.48–15.5 MBq (averaging ~ 7.4 MBq) of [^18^F]**1** in 100 μL of 10% EtOH in saline containing sodium ascorbate via tail vein injection. Immediately after injection, the mice were anesthetized using a 1 L oxygen flow of 3% isoflurane and imaged within 2–5 min of injection. Micro-PET image reconstruction was obtained with an OSEM3D algorithm without tissue attenuation correction. The micro-PET data was analyzed using Siemens Inveon Research Workplace, General Analysis software.

The micro-CT images were obtained on a MILabs VECTor^6^CT^UHR^OI unit (Houten, Utrecht, Netherlands) immediately after micro-PET for the purpose of anatomic/molecular data fusion. The accurate total body, full scan angle micro-CT images were acquired for ~ 8–10 min, and concurrent image reconstruction was achieved using a Hann projection filter algorithm at 100 µm voxel size. Reconstructed DICOM (digital imaging and communication in medicine) micro-CT images were created using PMOD 4.1 software and imported into the Siemens Inveon Research Workplace software for subsequent image fusion with micro-PET for the ROIs overlay to create TACs to access radioisotope uptake and distribution.

The second PET/CT scan followed the same imaging protocol as the first PET/CT scan except for the blocking agent (5 mg/kg) was administered intravenously 5 min prior to [^18^F]**1** injection. All mice were sacrificed 24 h following the completion of the second PET/CT scan, and their hearts and descending aortas were harvested and preserved in 4% paraformaldehyde in phosphate-buffered saline (PBS) solution until further analysis.

### Biodistribution studies

Seventeen-week-old male and female C57BL/6 mice were purchased to match the age of PET imaging mice. They were anesthetized in the anesthesia chamber using 3% isoflurane in oxygen and received 0.74–1.11 MBq of [^18^F]**1** in 100 μL of 5–10% EtOH in saline containing 0.5% sodium ascorbate. The injected mice were returned to their cages and free until the time of the study. At 10, 30, and 60 min after the [^18^F]**1** administration, each mouse (N = 4/time point) was anesthetized again and euthanized by cervical dislocation followed by cardiac puncture to take a blood sample. Then, the following organs were harvested: the brain, blood, heart, liver, lung, stomach, large intestine, small intestine, spleen, kidney, bone, muscle, liquinal LN, interscapular BAT, and tail. The radioactivity of each organ was counted by a PerkinElmer Wallac 1480 Wizard^3^ Gamma Counter (Waltham, MA). The whole radioactivity that went into the circulation of the mouse was calculated by subtracting the residual radioactivity in the tail from the injected radioactivity. Therefore, the biodistribution was calculated as an average of the percent injected dose per gram of tissue (%ID/g tissue) by dividing the measured radioactivity by the whole radioactivity that actually went into the body and the weight of the tissue.

### Tissue IHC

Terminally collected heart and aorta tissues were fixed by submerging them in 4% paraformaldehyde/PBS for 24 h. The tissue was cleaned of surrounding connective or adipose tissues and pictured to perform macroscopic observation for atherosclerosis development. Aorta was fragmented to represent PET-positive (or negative) regions, and each fragment was embedded in paraffin for subsequent microscopic histology analysis. CXCR3 and cell-type markers’ expression was assessed by IHC. To perform IHC, aorta Sects. (5 µm) were deparaffinized, dehydrated, and processed with heat-mediated antigen retrieval using citrate buffer (pH 6.0) followed by a blocking step (Protein block, ab64226, Abcam, Cambridge, UK). Sections were incubated overnight at 4°C with anti-CXCR3 antibody (NB100-56404, Novus Biologicals, Centennial, CO), anti-mouse CD3e antibody (MA5-17655, Invitrogen, Waltham, MA), anti-αSMA antibody (ab202368, Abcam), and anti-Mac3 antibody (108501, BioLegend, San Diego, CA) or with corresponding normal IgG as a negative control. Specific signals were amplified by the biotin-streptoavidin system or by using the Tyramide SuperBoost kit for corresponding host species of the antibody (Invitrogen), and then sections were counterstained by DAPI. After imaging, sections were counterstained with H&E to visualize tissue structure for the purpose of reference of IHC images.

## Supplementary Information

**Additional file 1. Figure S1**. (a) The semi-preparative HPLC of [^18^F]1 and (b) The analytical HPLC of [18F]1. Co-injection of nonradioactive standard 1 confirmed that the collected product was authentic.** Figure S2**. Competitive binding curves of cell-binding assays of compound 1 for (a) CXCR3A and (b) CXCR3B.** Figure S3**. Baseline PET and PET/CT images of two ApoE knockout mice and a C57BL/6 control mouse presented in Figure 3. Atherosclerotic lesions and brown adipose tissues are indicated by red arrows and blue arrows, respectively.Figure S4. NMR and Mass spectra(a) Methyl (S)-6-(4-(tert-butoxycarbonyl)-3-ethylpiperazin-1-yl)-5-chloronicotinate (4) (b) Methyl (S)-5-chloro-6-(3-ethylpiperazin-1-yl)nicotinate (5) (c) Methyl (S)-6-(4-(1-(tert-butoxycarbonyl)piperidin-4-yl)-3-ethylpiperazin-1-yl)-5-chloronicotinate (7) (d) tert-Butyl (S)-4-(4-(3-chloro-5-(1,3,4-oxadiazol-2-yl)pyridin-2-yl)-2-ethylpiperazin-1-yl)piperidine-1-carboxylate (8) (e) (S)-2-(5-Chloro-6-(3-ethyl-4-(piperidin-4-yl)piperazin-1-yl)pyridin-3-yl)-1,3,4-oxadiazole(9) (f)* N*-(4-Chloro-2-fluorobenzyl)-4-piperidinone (12) (g) Methyl (S)-5-chloro-6-(4-(1-(4-chloro-2-fluorobenzyl)piperidin-4-yl)-3-ethylpiperazin-1-yl)nicotinate (13) (h) (S)-2-(5-chloro-6-(4-(1-(4-chloro-2-fluorobenzyl)piperidin-4-yl)-3-ethylpiperazin-1-yl)pyridin-3-yl)-1,3,4-oxadiazole (1).

## Data Availability

All data presented in this paper is available from the corresponding author upon a reasonable request.

## Abbreviations

PET: Positron emission tomography
CT: Computed tomography
HEK: Human embryonic kidney
ApoE: Apolipoprotein E
KO: Knockout
TAC: Time-activity curve
SUV: Standard uptake value
IHC: Immunohistochemistry
RCY: Radiochemical yield
RCP: Radiochemical purity
EOS: End of synthesis
BAT: Brown adipose tissue
Th1 cells: Type 1 helper T cells
Nk cells: Natural killer cells
CAM: Computer-aided modeling
TFA: Trifluoroacetic acid
HCl: Hydrochloride
EtOH: Ethanol
SPE: Solid phase extraction
RCC: Radiochemical conversion
TLC: Thin-layer chromatography
HBr: Bromic acid
AcOH: Acetic acid
DMF: *N*,*N*-Dimethylformamide
MeCN: Acetonitrile
Na_2_SO_4_: Sodium sulfate
K_2_CO_3_: Potassium carbonate
DIPEA: Diisopropylethylamine
NaBH_3_CN: Sodium cyanoborohydride
MeOH: Methanol
NEt_3_: Triethylamine
ROI: Region of interest
AUC: Area under the curve
LN: Lymph node
%ID/g tissue: Percent injected dose per gram tissue
H&E: Hematoxylin and eosin
EtOAc: Ethyl acetate
DCM: Dichloromethane
ESI: Electrospray ionization
APCI: Atmospheric pressure chemical ionization
MS: Mass spectra
HRMS: High resolution mass spectra
NaHCO_3_: Sodium bicarbonate
RT: Room temperature
DCE: 1,2-Dichloroethane
MgSO_4_: Magnesium sulfate
DMEM media: Dulbecco’s Modified Eagle media
FBS: Fetal bovine serum
K_i_: Equilibrium inhibition constant
FOV: Field of view
PBS: Phosphate-buffered saline
αSMA: α-Smooth muscle actin
SMC: Smooth muscle cell
