# Supplementary material for: Synthesis and preclinical evaluation of a novel fluorine-18 labeled small-molecule PET radiotracer for imaging of CXCR3 receptor in mouse models of atherosclerosis
Source: EJNMMI Res. 2023 Jul 13;13:67. doi: 10.1186/s13550-023-01017-x (PMC10338423; doi:10.1186/s13550-023-01017-x)
Supplement: Supplementary file 1 — Additional file 1. Figure S1. (a) The semi-preparative HPLC of [18F]1 and (b) The analytical HPLC of [18F]1. Co-injection of nonradioactive standard 1 confirmed that the collected product was authentic. Figure S2. Competitive binding curves of cell-binding assays of compound 1 for (a) CXCR3A and (b) CXCR3B. Figure S3. Baseline PET and PET/CT images of two ApoE knockout mice and a C57BL/6 control mouse presented in Figure 3. Atherosclerotic lesions and brown adipose tissues are indicated by red arrows and blue arrows, respectively.Figure S4. NMR and Mass spectra(a) Methyl (S)-6-(4-(tert-butoxycarbonyl)-3-ethylpiperazin-1-yl)-5-chloronicotinate (4) (b) Methyl (S)-5-chloro-6-(3-ethylpiperazin-1-yl)nicotinate (5) (c) Methyl (S)-6-(4-(1-(tert-butoxycarbonyl)piperidin-4-yl)-3-ethylpiperazin-1-yl)-5-chloronicotinate (7) (d) tert-Butyl (S)-4-(4-(3-chloro-5-(1,3,4-oxadiazol-2-yl)pyridin-2-yl)-2-ethylpiperazin-1-yl)piperidine-1-carboxylate (8) (e) (S)-2-(5-Chloro-6-(3-ethyl-4-(piperidin-4-yl)piperazin-1-yl)pyridin-3-yl)-1,3,4-oxadiazole(9) (f) N-(4-Chloro-2-fluorobenzyl)-4-piperidinone (12) (g) Methyl (S)-5-chloro-6-(4-(1-(4-chloro-2-fluorobenzyl)piperidin-4-yl)-3-ethylpiperazin-1-yl)nicotinate (13) (h) (S)-2-(5-chloro-6-(4-(1-(4-chloro-2-fluorobenzyl)piperidin-4-yl)-3-ethylpiperazin-1-yl)pyridin-3-yl)-1,3,4-oxadiazole (1). [file 13550_2023_1017_MOESM1_ESM.pdf]

Supplementary Information

Synthesis and preclinical evaluation of a novel fluorine-18 labeled small-molecule PET radiotracer for imaging of CXCR3 receptor in mouse models of atherosclerosis

Santosh R. Alluri[1†], Yusuke Higashi[2], Ashley Berendzen[3], Laurel A. Grisanti[4], Lisa D. Watkinson[3], Kamlendra Singh[5], Timothy J. Hoffman[3], Terry Carmack[3], Elizabeth A. Devanny[3], Miles Tanner[4], Kun-Eek Kil[1,6*]

1. University of Missouri Research Reactor, University of Missouri, Columbia, Missouri 65211, USA
2. Department of Medicine, Tulane University School of Medicine, New Orleans, Louisiana 70112, USA
3. Research Service, Harry S. Truman Memorial Veterans' Hospital, Columbia, Missouri 65201, USA
4. Department of Biomedical Sciences, College of Veterinary Medicine, University of Missouri, Columbia, Missouri 65211, USA
5. Bond Life Sciences Center, University of Missouri, Columbia, Missouri 65211, USA
6. Department of Veterinary Medicine and Surgery, College of Veterinary Medicine, University of Missouri, Columbia, Missouri 65211, USA

* For correspondence:

Kun-Eek Kil: University of Missouri Research Reactor, University of Missouri, 1513 Research Park Drive, Columbia, Missouri 65211, USA, kilk@missouri.edu, 573-884-7885

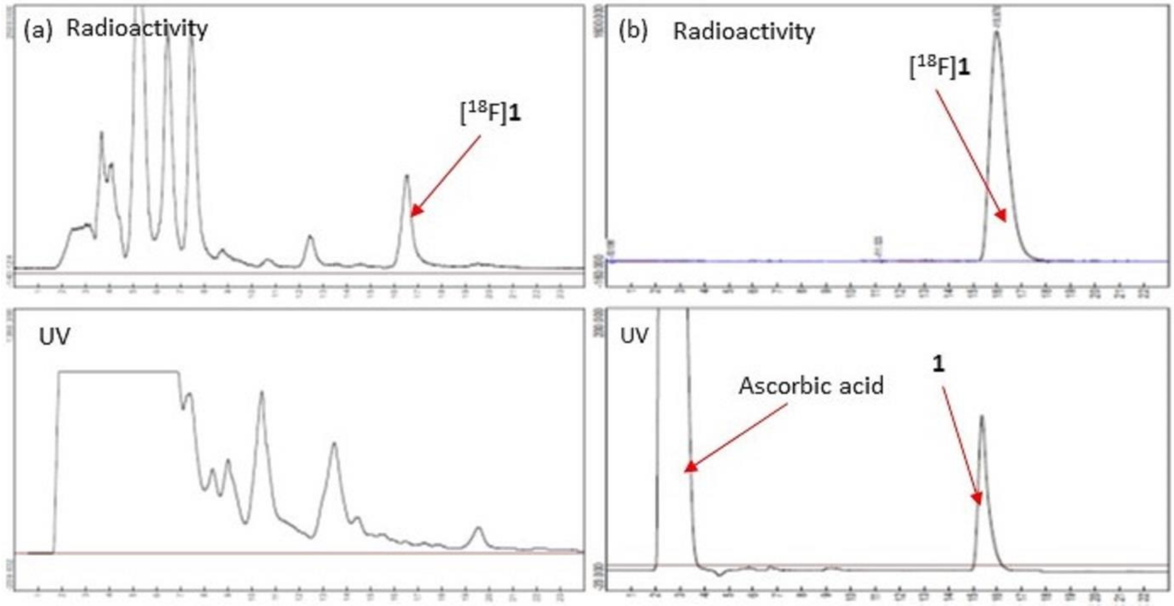

Figure S1. (a) The semi-preparative HPLC of [^{18}F]1 and (b) The analytical HPLC of [^{18}F]1. Co-injection of nonradioactive standard 1 confirmed that the collected product was authentic.

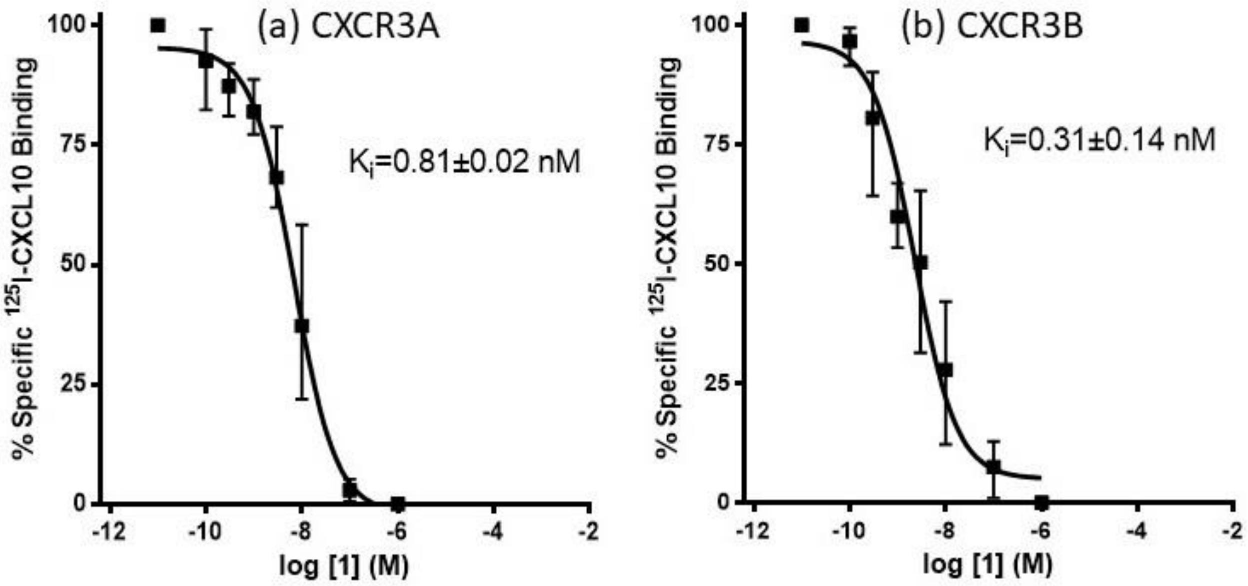

Figure S2. Competitive binding curves of cell-binding assays of compound 1 for (a) CXCR3A and (b) CXCR3B

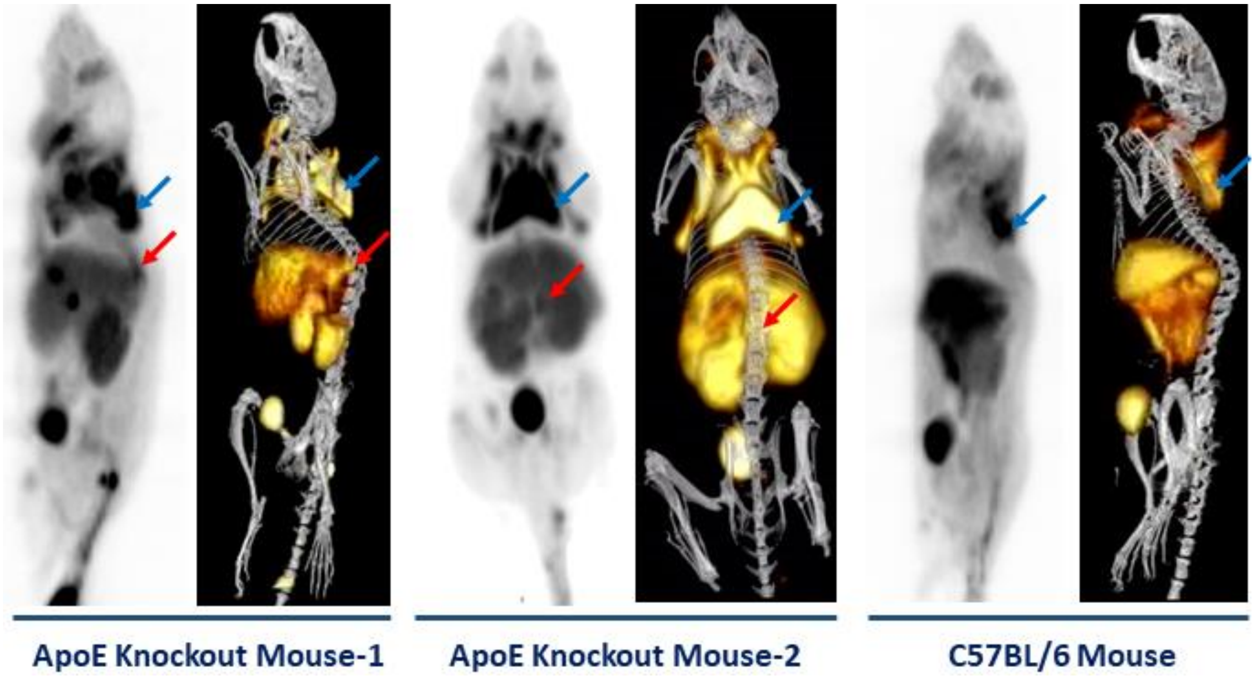

Figure S3. Baseline PET and PET/CT images of two ApoE knockout mice and a C57BL/6 control mouse presented in Figure 3. Atherosclerotic lesions and brown adipose tissues are indicated by red arrows and blue arrows, respectively.

Figure S4. NMR and Mass spectra

(a) Methyl (S)-6-(4-(tert-butoxycarbonyl)-3-ethylpiperazin-1-yl)-5-chloronicotinate (4)

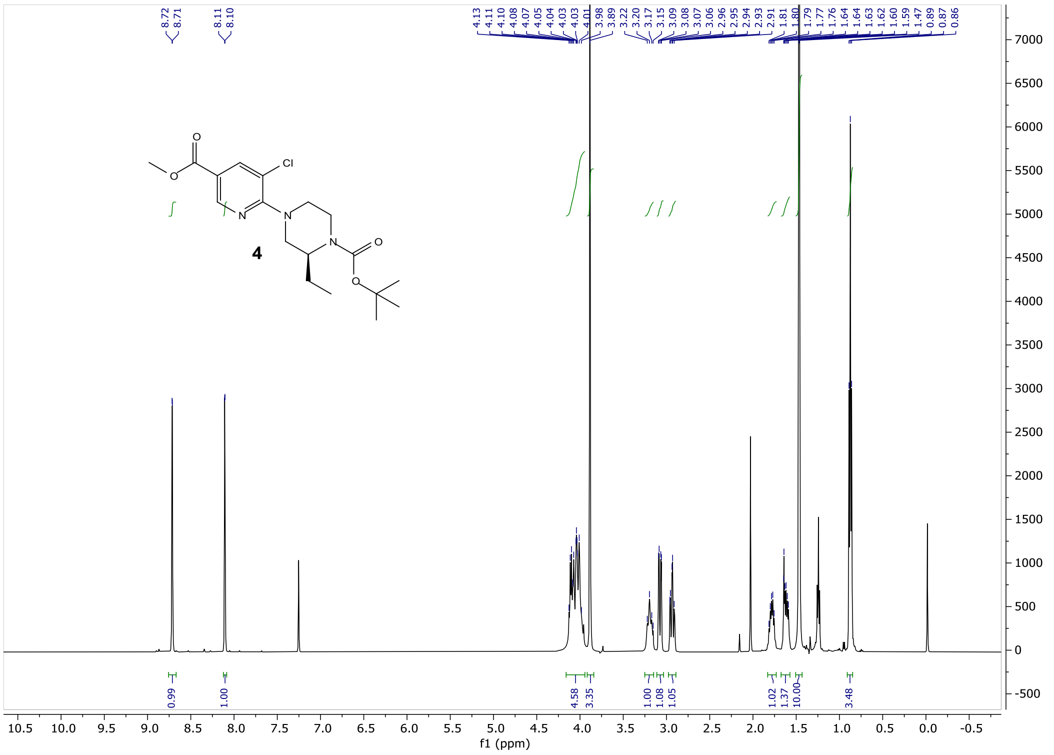

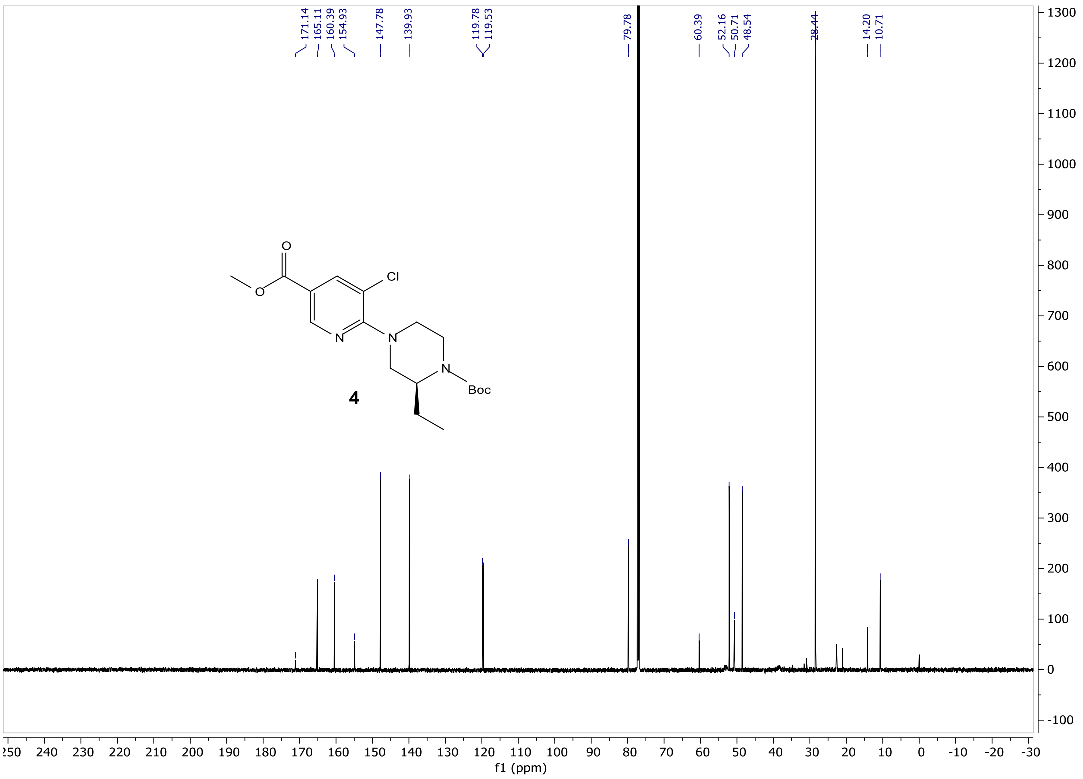

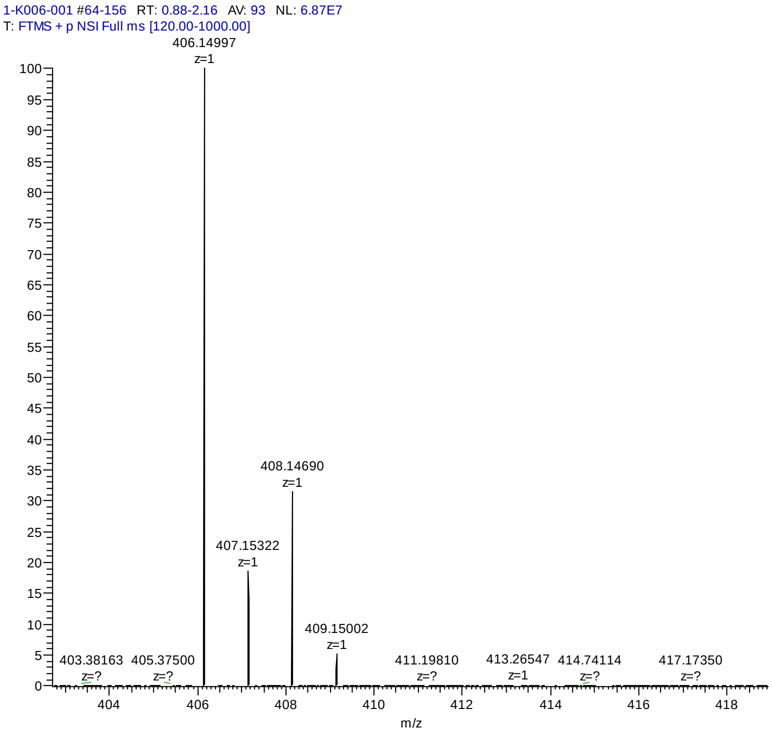

(b) Methyl (S)-5-chloro-6-(3-ethylpiperazin-1-yl)nicotinate (5)

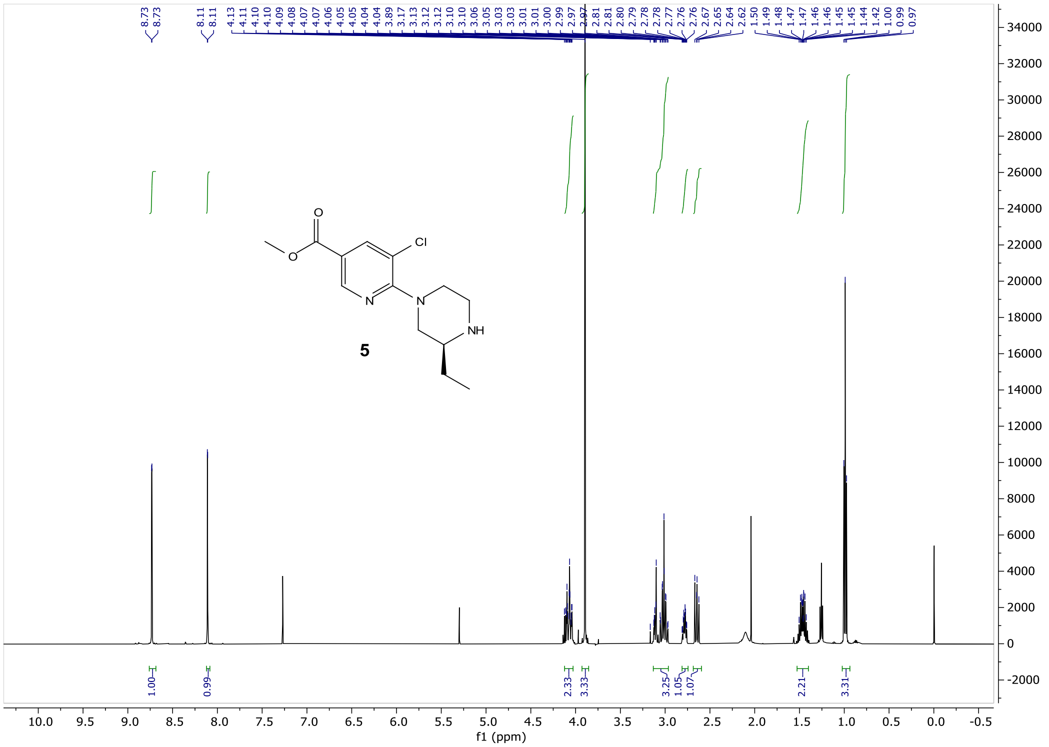

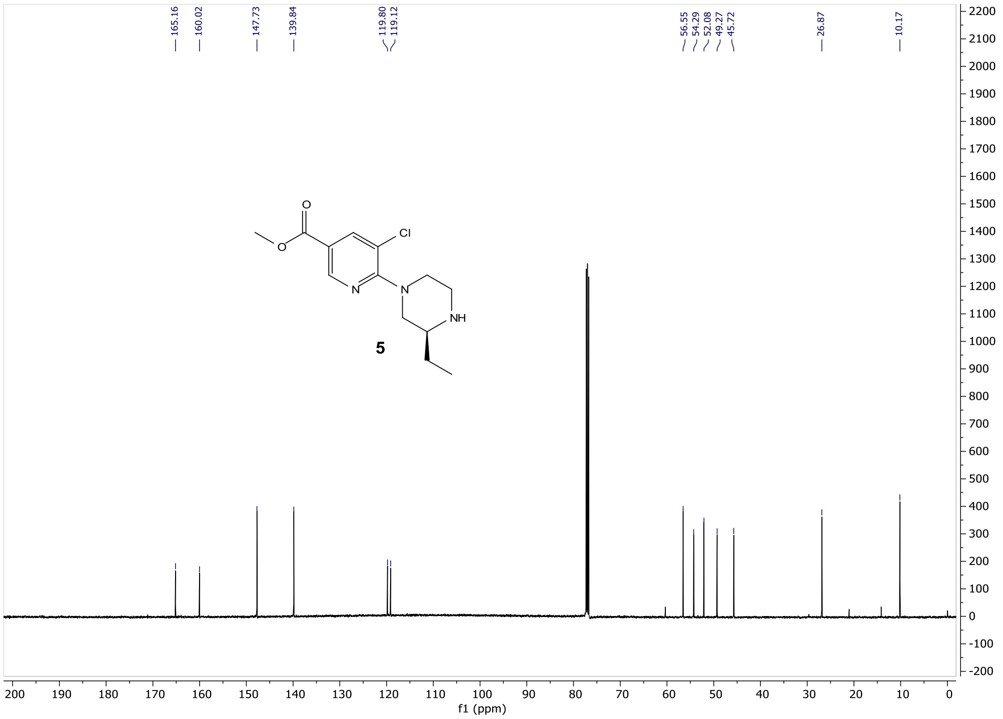
165.16
160.02
147.73
139.84
119.80
119.12
56.55
54.29
52.08
49.27
45.72
26.87
10.17
O
O
Cl
N
N
NH
5
200 190 180 170 160 150 140 130 120 110 100 90 80 70 60 50 40 30 20 10 0
f1 (ppm)
2200 2100 2000 1900 1800 1700 1600 1500 1400 1300 1200 1100 1000 900 800 700 600 500 400 300 200 100 0 -100 -200

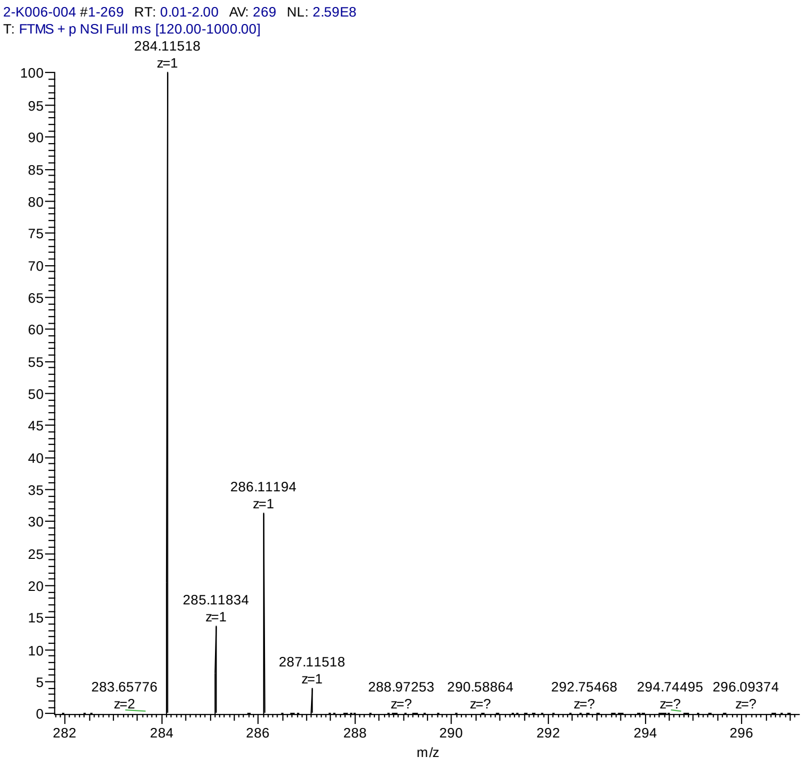
2-K006-004 #1-269 RT: 0.01-2.00 AV: 269 NL: 2.59E8
T: FTMS + p NSI Full ms [120.00-1000.00]
284.11518
z=1
286.11194
z=1
285.11834
z=1
287.11518
z=1
283.65776
z=2
288.97253
z=?
290.58864
z=?
292.75468
z=?
294.74495
z=?
296.09374
z=?
100 95 90 85 80 75 70 65 60 55 50 45 40 35 30 25 20 15 10 5 0
282 284 286 288 290 292 294 296
m/z

(c) Methyl (S)-6-(4-(1-(tert-butoxycarbonyl)piperidin-4-yl)-3-ethylpiperazin-1-yl)-5-chloronicotinate (7)

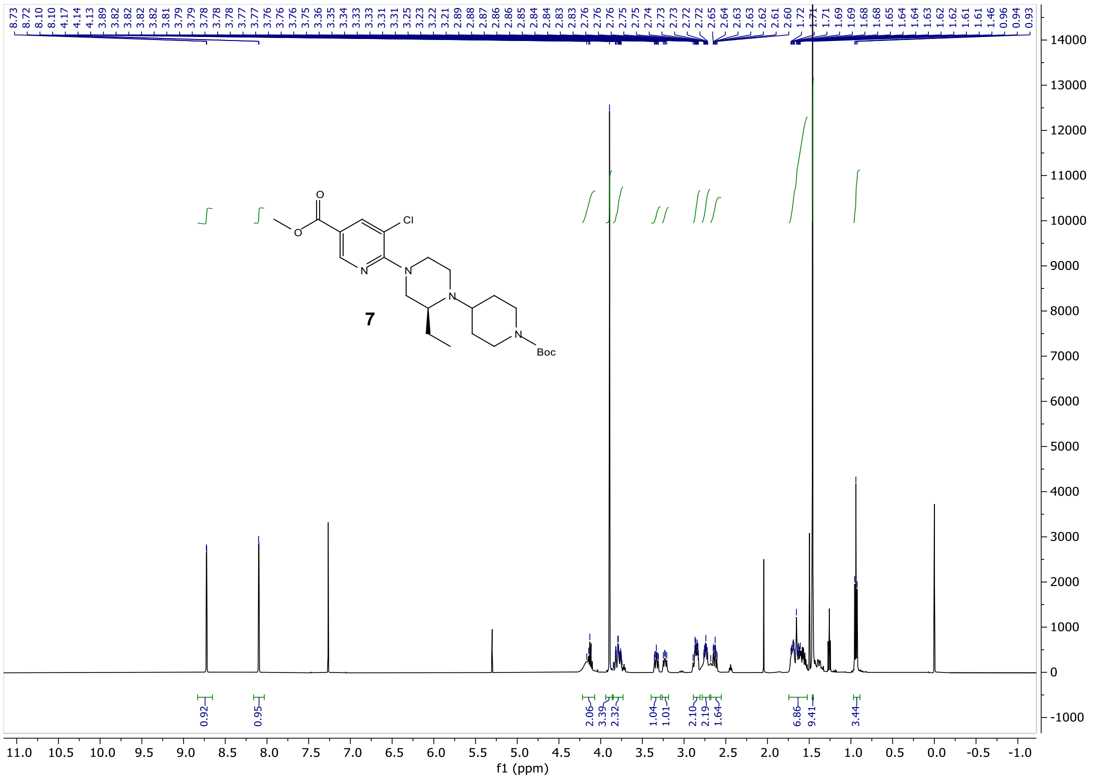

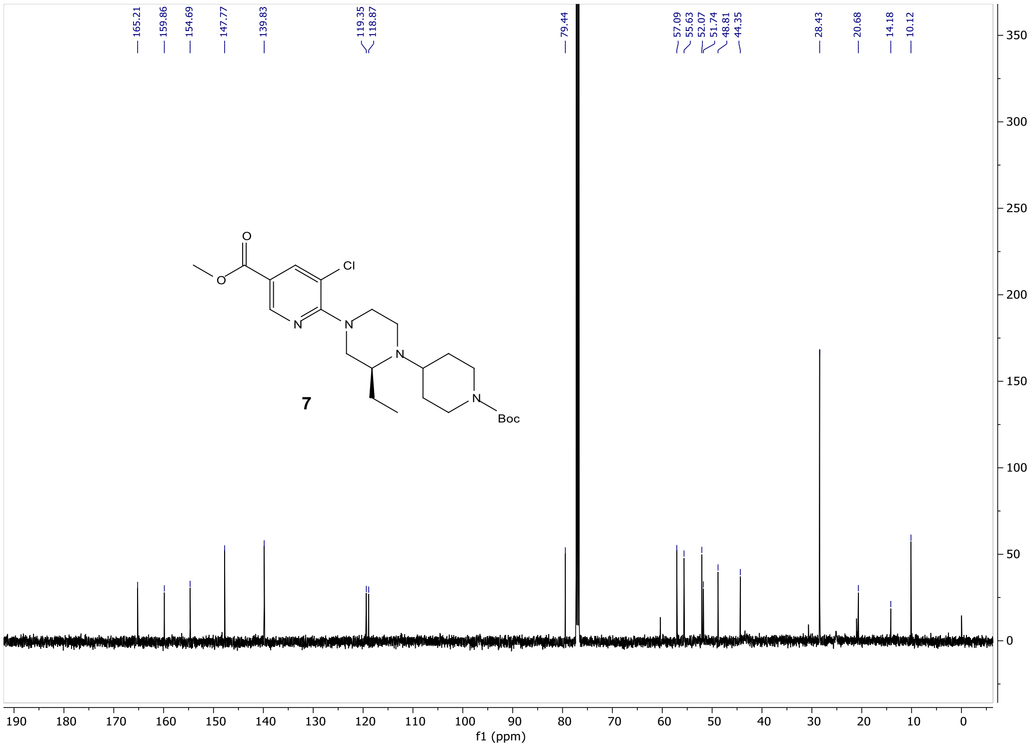

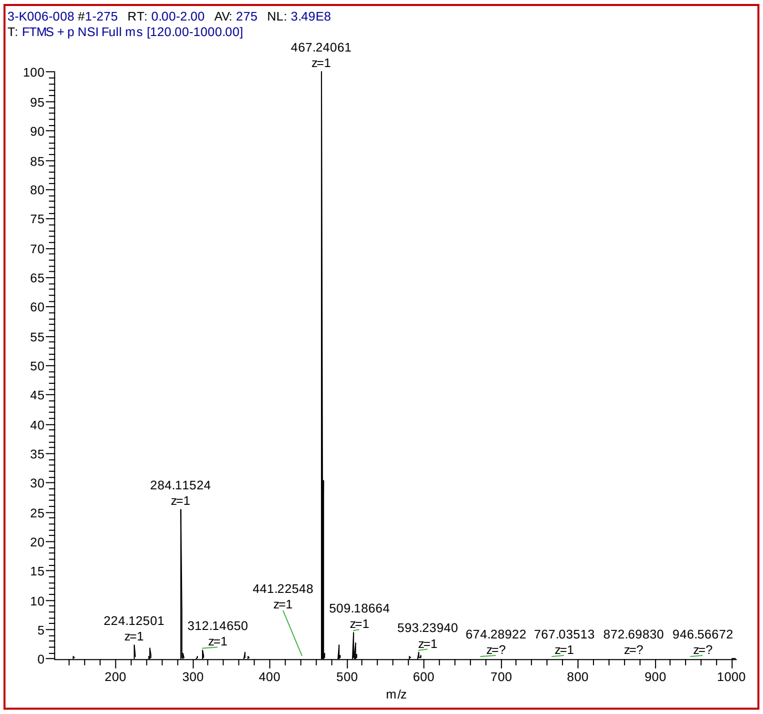

(d) tert-Butyl (S)-4-(4-(3-chloro-5-(1,3,4-oxadiazol-2-yl)pyridin-2-yl)-2-ethylpiperazin-1-yl)piperidine-1-carboxylate (8)

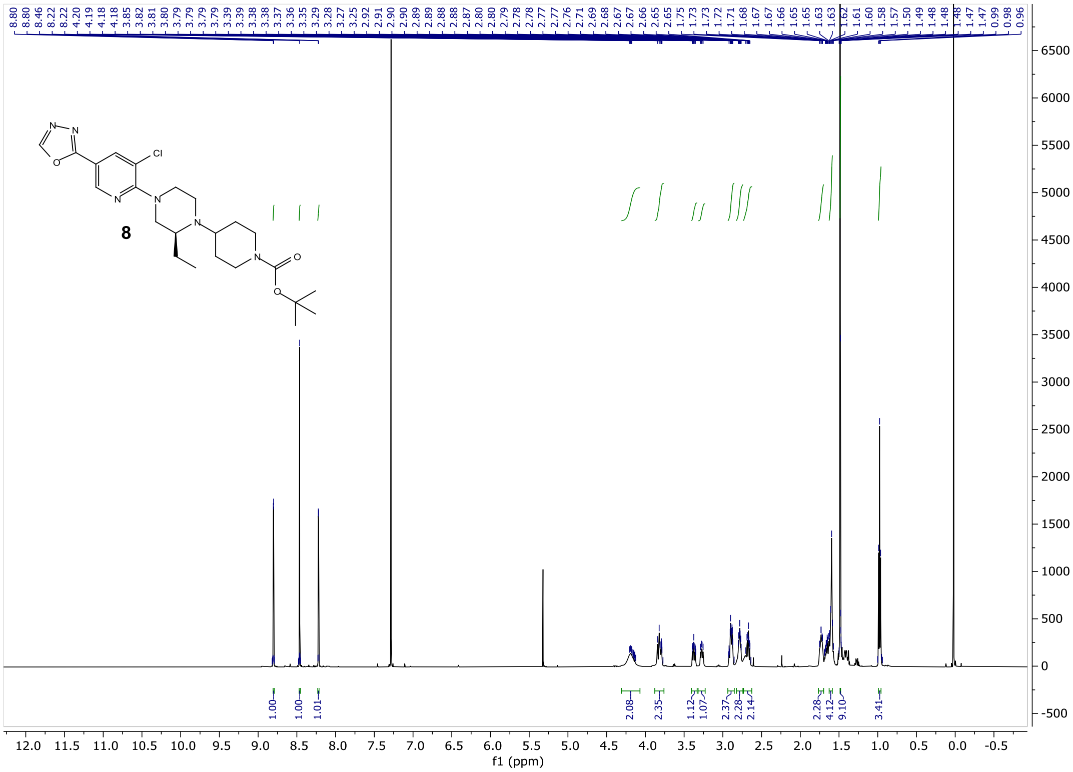

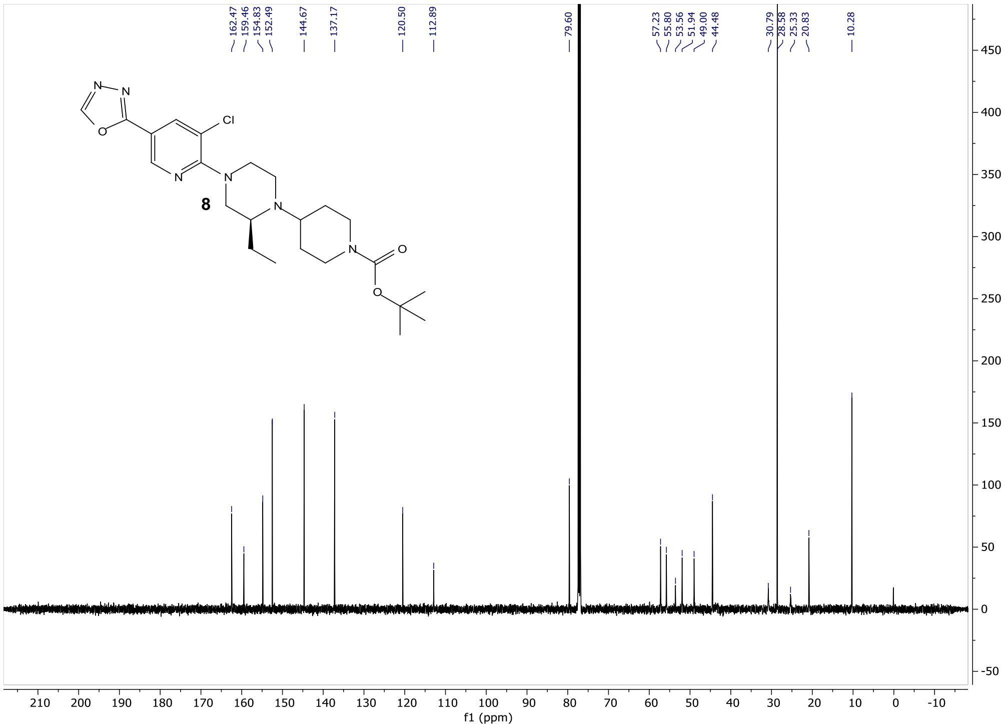

162.47
159.46
154.83
152.49
144.67
137.17
120.50
112.89
79.60
57.23
55.80
53.56
51.94
49.00
44.48
30.79
28.58
25.33
20.83
10.28
Cl
N
O
8
f1 (ppm)

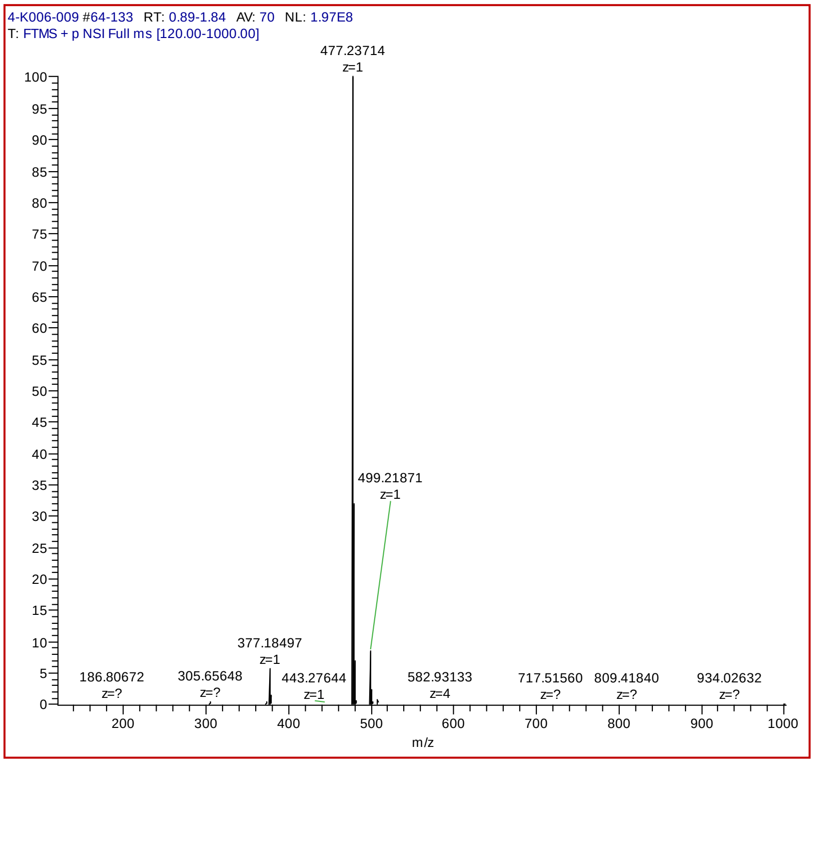

4-K006-009 #64-133 RT: 0.89-1.84 AV: 70 NL: 1.97E8
T: FTMS + p NSI Full ms [120.00-1000.00]
477.23714
z=1
499.21871
z=1
377.18497
z=1
186.80672
z=?
305.65648
z=?
443.27644
z=1
582.93133
z=4
717.51560
z=?
809.41840
z=?
934.02632
z=?
m/z

(e) (S)-2-(5-Chloro-6-(3-ethyl-4-(piperidin-4-yl)piperazin-1-yl)pyridin-3-yl)-1,3,4-oxadiazole (9)

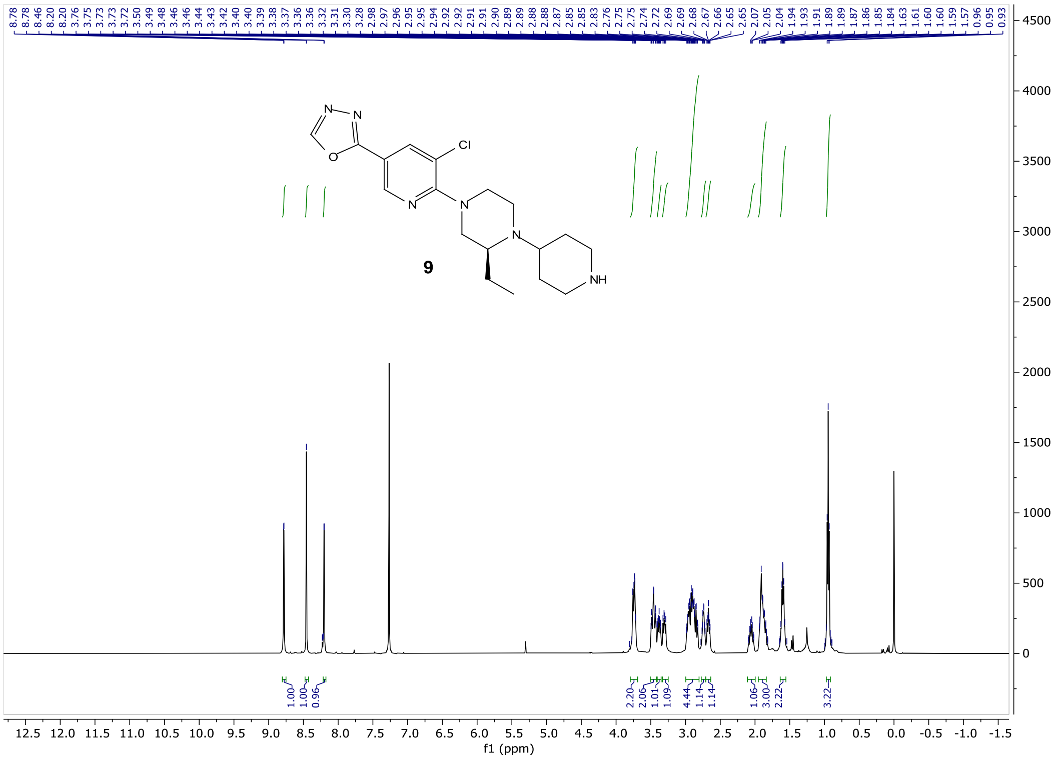

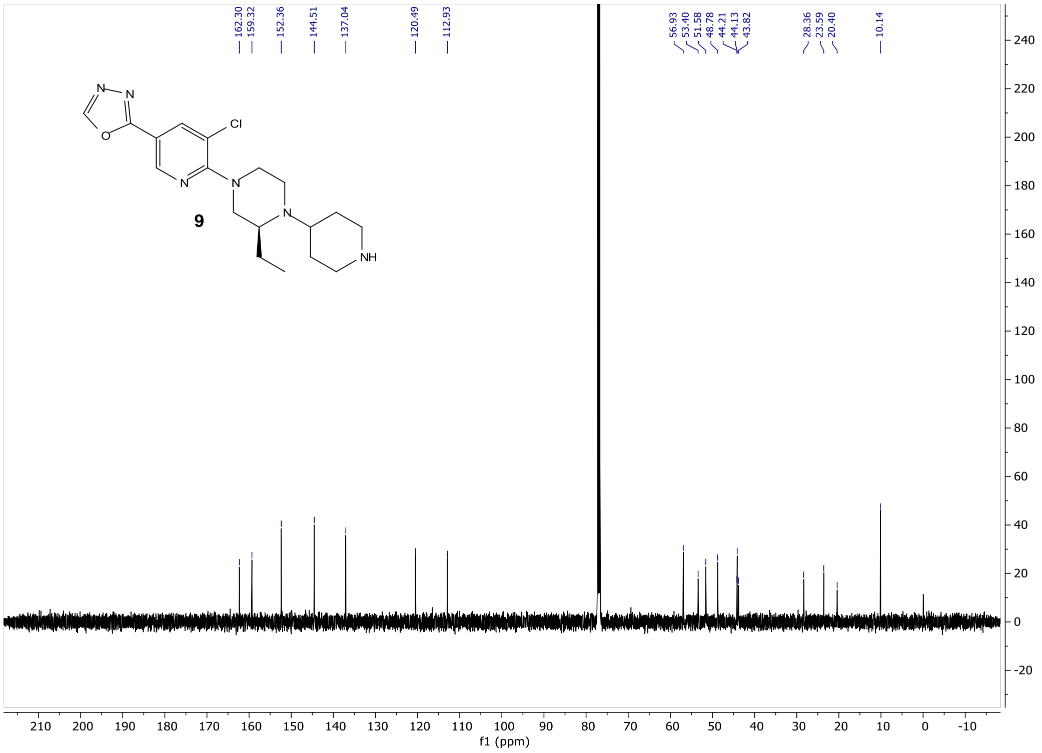

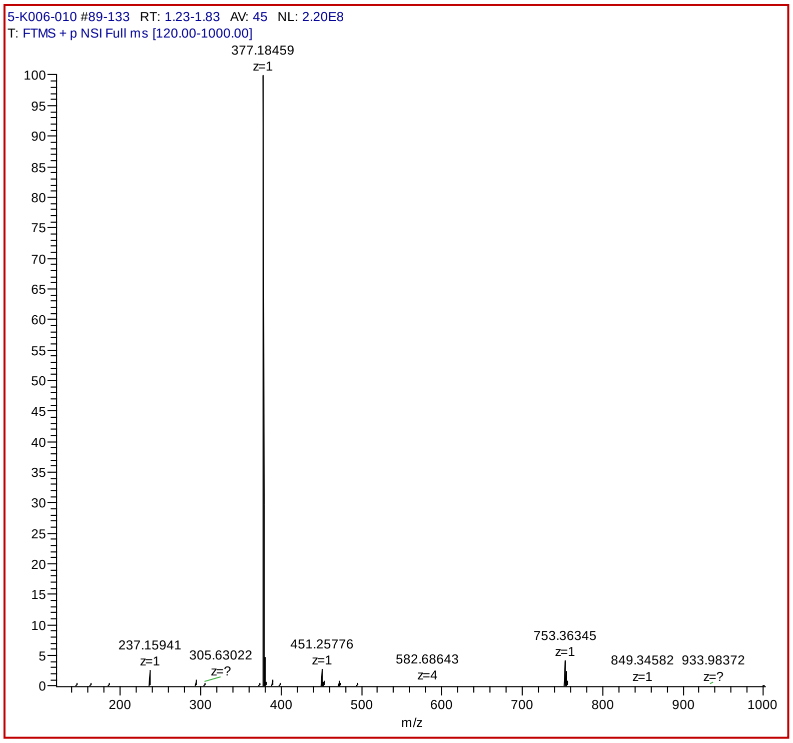

(f) *N*-(4-Chloro-2-fluorobenzyl)-4-piperidinone (12)

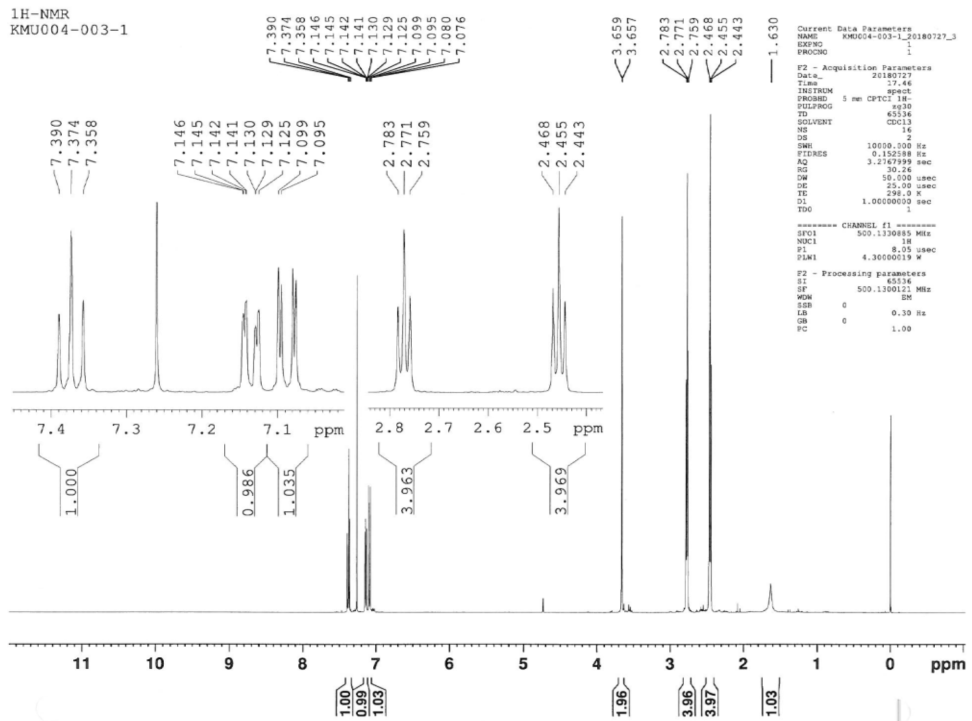

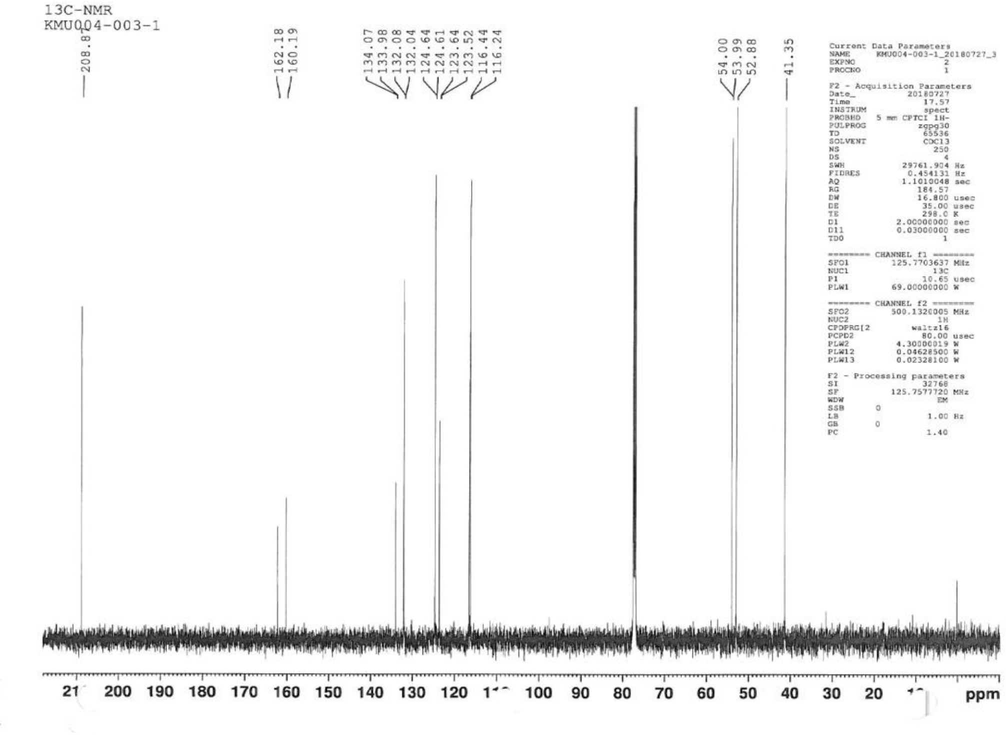
13C-NMR
KMU004-003-1
208.8
162.18
160.19
134.07
133.98
132.08
132.04
124.64
124.61
123.64
123.52
116.44
116.24
54.00
53.99
52.88
41.35
ppm

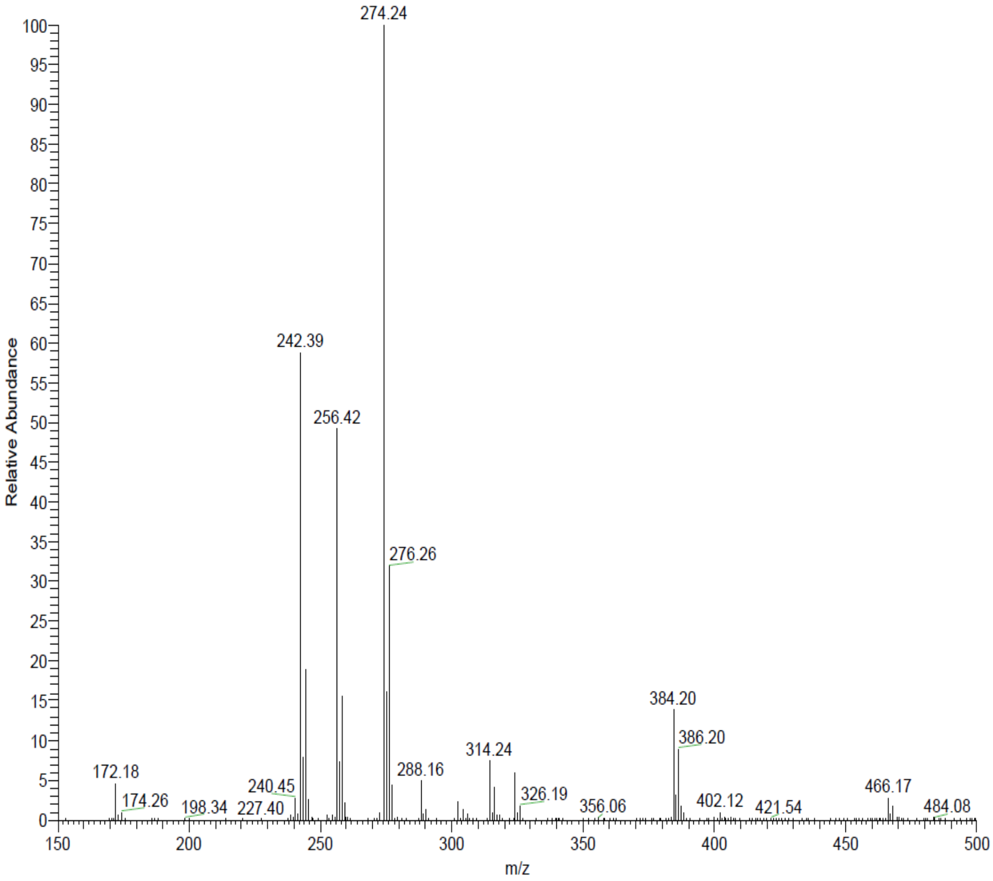
Relative Abundance
m/z
274.24
242.39
256.42
276.26
384.20
386.20
314.24
172.18
288.16
240.45
326.19
466.17
174.26
198.34
227.40
356.06
402.12
421.54
484.08

(g) Methyl (S)-5-chloro-6-(4-(1-(4-chloro-2-fluorobenzyl)piperidin-4-yl)-3-ethylpiperazin-1-yl)nicotinate (13)

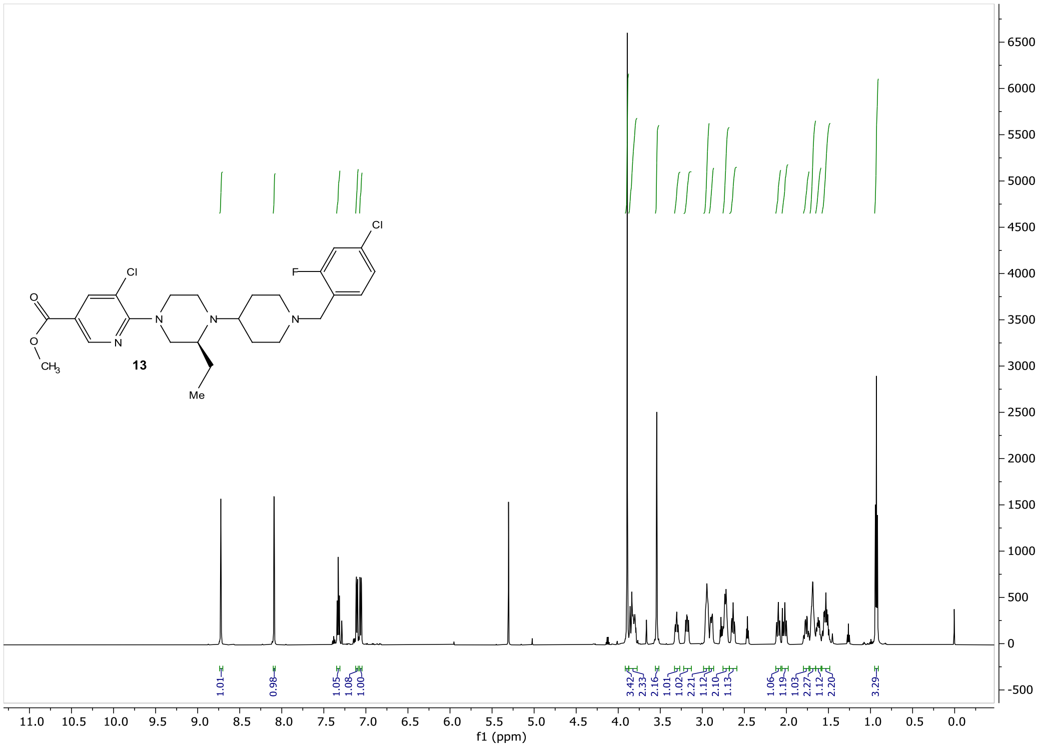

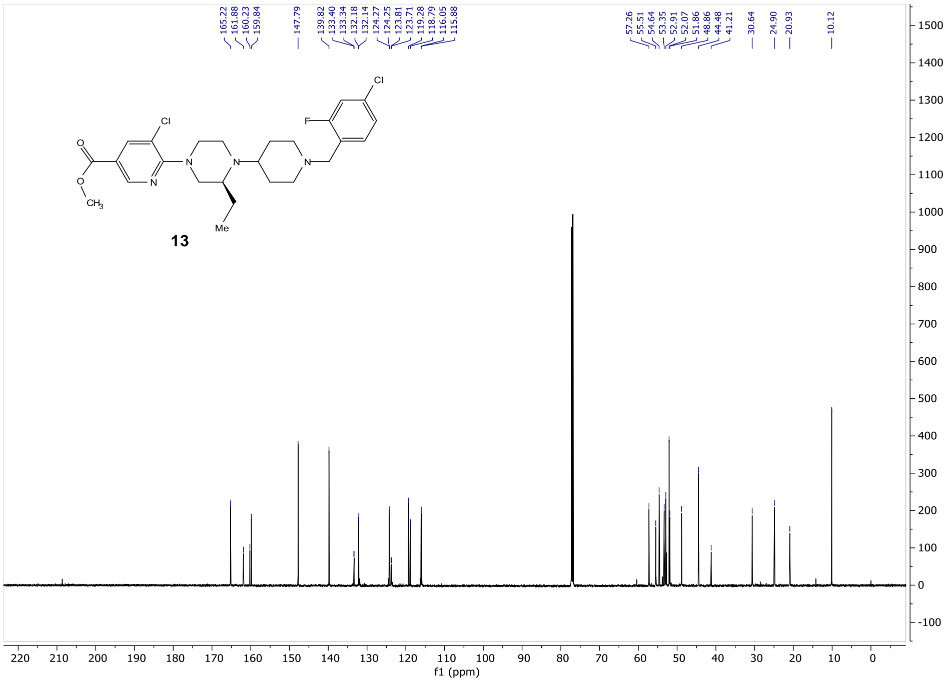

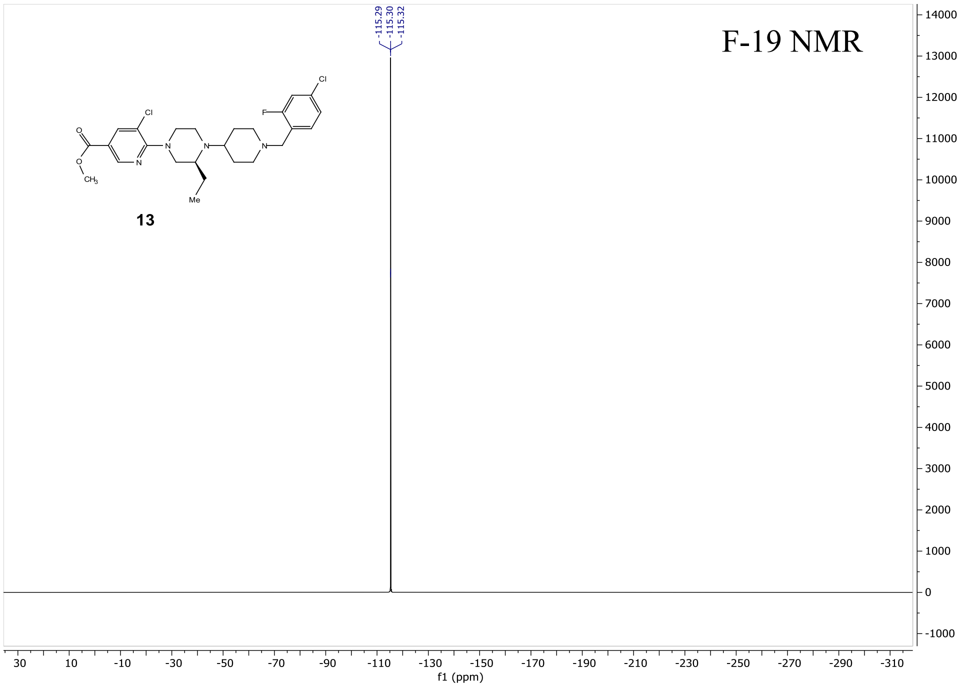

F-19 NMR
-115.29
-115.30
-115.32
13
Cl
Cl
F
O
O
CH3
N
N
N
N
Me
f1 (ppm)

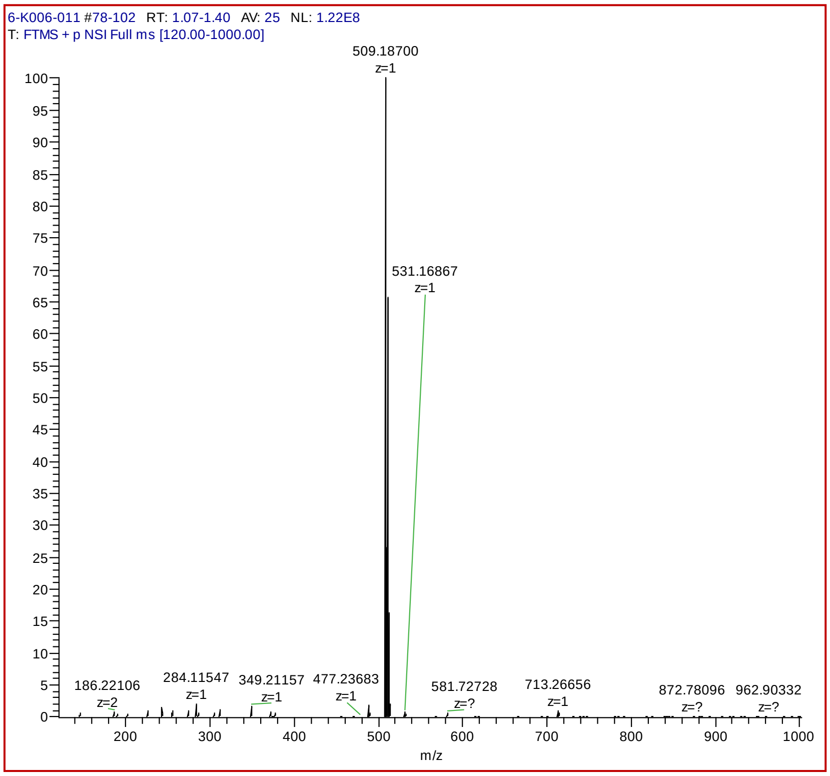

6-K006-011 #78-102 RT: 1.07-1.40 AV: 25 NL: 1.22E8
T: FTMS + p NSI Full ms [120.00-1000.00]
509.18700
z=1
531.16867
z=1
186.22106
z=2
284.11547
z=1
349.21157
z=1
477.23683
z=1
581.72728
z=?
713.26656
z=1
872.78096
z=?
962.90332
z=?
m/z

(h) (S)-2-(5-chloro-6-(4-(1-(4-chloro-2-fluorobenzyl)piperidin-4-yl)-3-ethylpiperazin-1-yl)pyridin-3-yl)-1,3,4-oxadiazole (1)

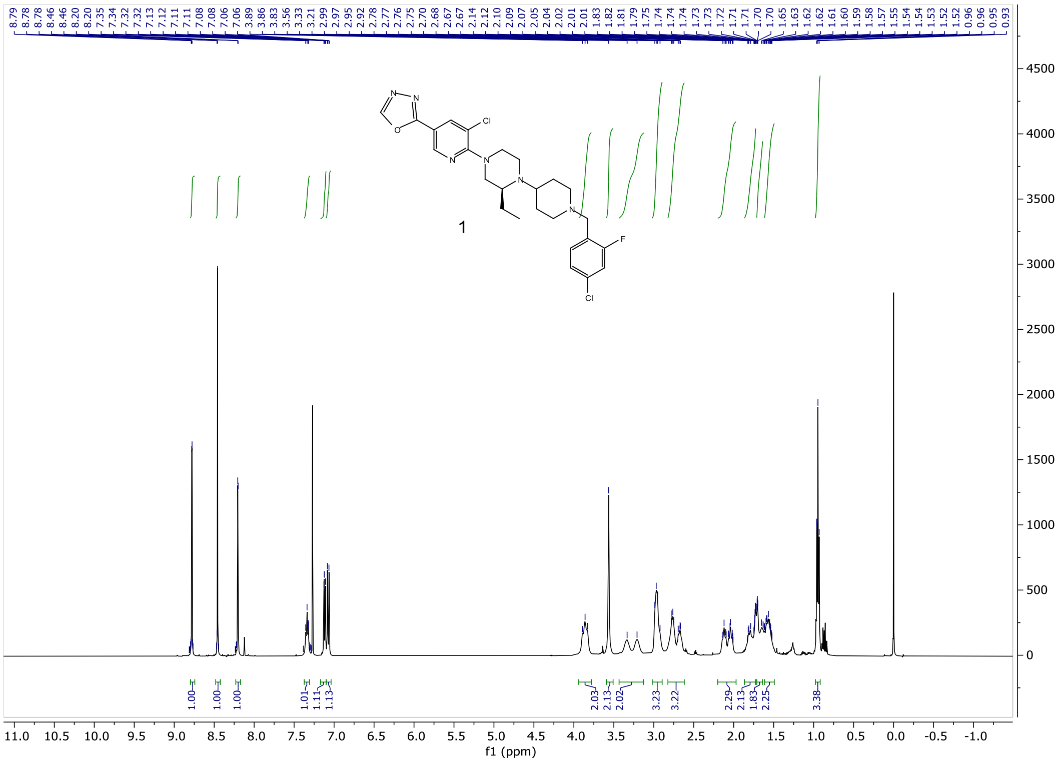

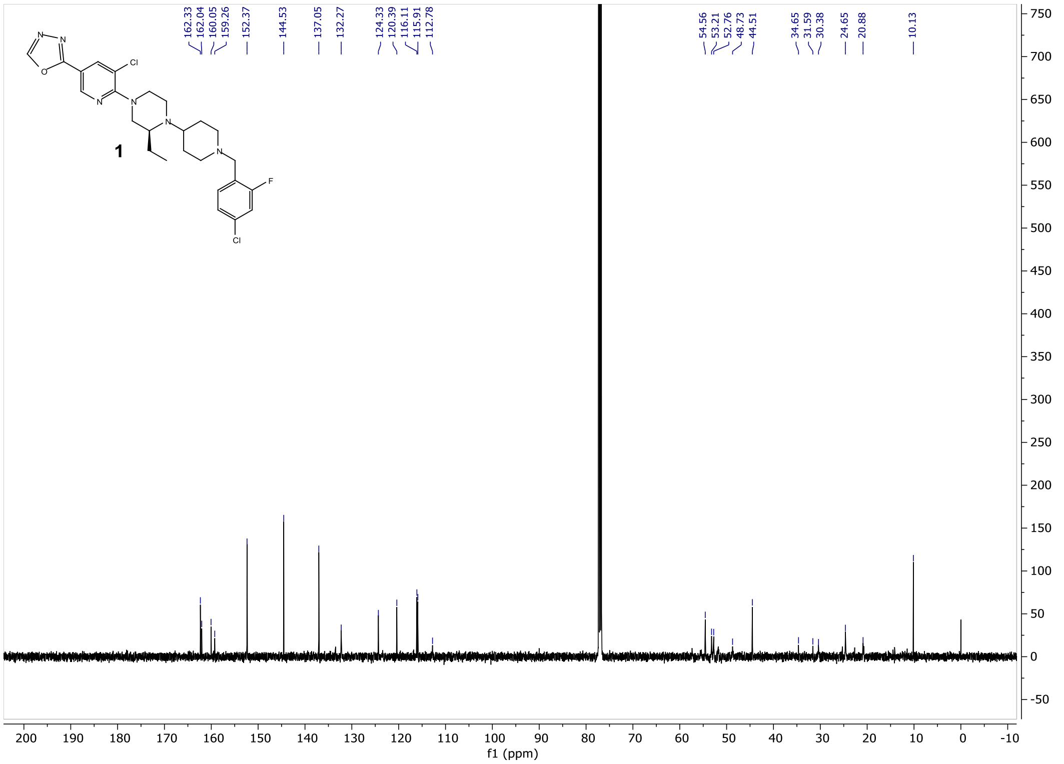

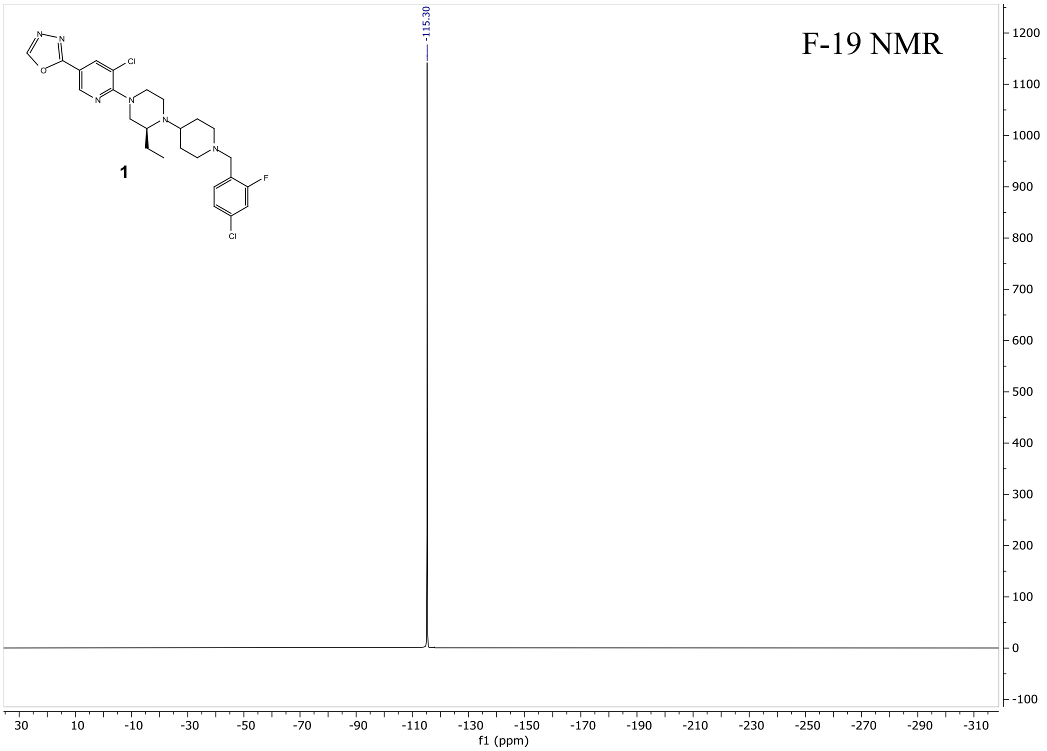
F-19 NMR
-115.30
1
f1 (ppm)

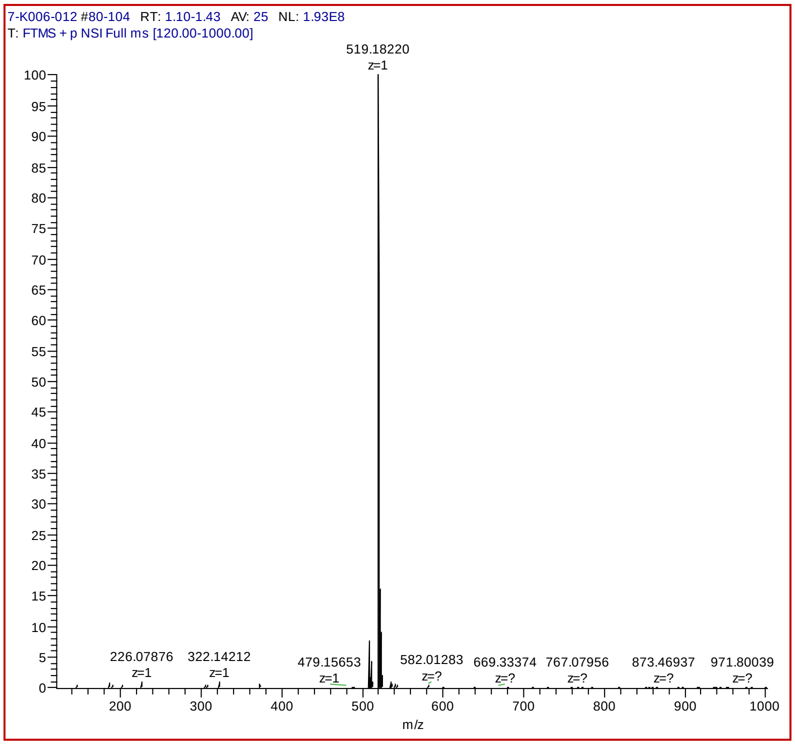
7-K006-012 #80-104 RT: 1.10-1.43 AV: 25 NL: 1.93E8
T: FTMS + p NSI Full ms [120.00-1000.00]
519.18220
z=1
226.07876
z=1
322.14212
z=1
479.15653
z=1
582.01283
z=?
669.33374
z=?
767.07956
z=?
873.46937
z=?
971.80039
z=?
m/z
